# Gut microbiota-epigenetic interactions in systemic aging: mechanistic drivers for endocrine and reproductive network remodeling and therapeutic modulation

**DOI:** 10.3389/fragi.2026.1826382

**Published:** 2026-06-01

**Authors:** Christopher Birigwa, Qiang Tong, Bing Qu, Teng Zuo, Wenzheng Yuan, Jing Xiong, Jianfei Luo

**Affiliations:** 1 Department of Gastrointestinal Surgery, Renmin Hospital of Wuhan University, Wuhan, China; 2 School of Medicine, Wuhan University of Science and Technology, Wuhan, China

**Keywords:** chromatin remodeling, endocrine aging, epigenetic regulation, gut microbiota, host–microbiome interactions, metabolic signaling, microbiota-derived metabolites, multi-omics

## Abstract

Researchers now see aging as a process shaped by the interactions among metabolism, epigenetics, and hormones. Recent studies suggest that gut microbes play an important role in this system by making metabolites that can affect gene expression and chromatin structure. Still, it is not fully clear how gut microbes and the body influence each other as we age, since both are constantly changing. This review brings together current research on how metabolites from gut microbes—such as short-chain fatty acids, bile acids, tryptophan derivatives, and polyamines—affect the body’s epigenetic machinery through processes such as DNA methylation, histone modifications, and chromatin remodeling. We examine evidence from cell studies, animal experiments, and human research to assess the strength of the links and distinguish direct effects on chromatin from indirect metabolic or gene-expression changes. We focus especially on endocrine and reproductive organs, such as the hypothalamus, pancreas, liver, fat tissue, and cells that support the gonads, where signals from gut microbes overlap with hormonal control and metabolism. In these tissues, microbial metabolites influence key pathways related to inflammation, mitochondria, and nutrient sensing, but there is still little direct evidence in humans. The review also points out differences between lab models and what is observed in patients, highlighting the need for further work to apply these findings in real-world settings. Interactions between gut microbes and epigenetics form a two-way link between metabolism, immunity, and aging of the endocrine system. While more evidence shows that microbial metabolites can shape gene activity and epigenetic patterns, most of what we know comes from animal studies rather than direct tests in people. Moving forward, researchers will need to use broad, long-term studies that combine different types of data to figure out cause and effect and which tissues are involved. Understanding this system better could help create new biomarkers and treatments to influence aging by targeting the microbiome and its effects on epigenetics.

## 1 Introduction

**FIGURE 1:**
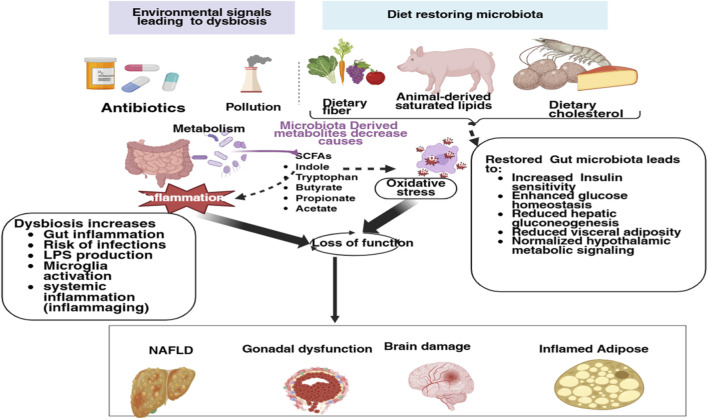
Functional consequences of dysbiosis and microbiota restoration on metabolic and endocrine aging pathways. The diagram illustrates the bidirectional influence of environmental exposures and dietary patterns on gut microbial composition and, consequently, host physiology *via* microbiota-derived metabolites. The diagram shows two opposing pathways. On the left, environmental stressors (antibiotics, pollution, low-fiber diets) cause dysbiosis, reducing beneficial microbial metabolites (SCFAs, indoles, tryptophan). This leads to oxidative stress, inflammaging (LPS, microglial activation, systemic inflammation), and organ dysfunction (NAFLD, gonadal failure, brain damage, inflamed fat). On the right, dietary fiber restores the microbiota, increasing metabolite production, which improves insulin sensitivity, glucose homeostasis, liver function, and hypothalamic signaling. The bottom bar indicates the evidence translation spectrum from cellular models to human trials.

Although global life expectancy is rising, the prevalence of age-related metabolic, endocrine, and reproductive disorders is also increasing. This trend is not fully explained by chronological age alone (Borrego-Ruiz and Borrego, 2024). Aging is now seen as a result of disrupted inter-organ communication, particularly through metabolic, immune, and hormonal signals. The gut microbiota, a complex community of intestinal microorganisms, has emerged as a central regulator of this crosstalk (Figure 1). It generates a wide range of bioactive metabolites and signaling molecules. These enter the circulation and affect distant tissues (Protachevicz et al., 2026; Gyriki et al., 2025). Evidence from cell and animal studies shows that microbial metabolites can modify chromatin states and influence gene expression programs (Morandini et al., 2023; Izadi et al., 2024). With age, the composition and metabolic activity of the gut microbiota change in predictable ways. Production of short-chain fatty acids (SCFAs) declines. Bile acid metabolism is altered, and amino acid catabolism becomes disrupted (Melnitskaia et al., 2024; Conway and A Duggal, 2021; Lim and Nam, 2023). These changes are linked to increased intestinal permeability, chronic low-grade inflammation (called “inflammaging”), and hormonal dysregulation. These are recognized hallmarks of aging. Many microbial metabolites regulate host epigenetic machinery. They do so by modulating histone deacetylases, DNA and histone methyltransferases, sirtuin pathways, and nuclear receptor-associated chromatin remodeling complexes (Best et al., 2025; Zhou et al., 2021). Through these mechanisms, microbial signals affect transcriptional stability in hormone-sensitive tissues. These tissues govern energy balance, stress responses, and reproduction. This suggests that changes in the gut microbiota are active contributors to aging rather than mere passive consequences (Missong et al., 2024; Wu Y. et al., 2021).

The interactions among the gut microbiota, epigenetic regulation, and aging of endocrine and reproductive systems form a fast-moving research field. It is well established that the microbiota influences aging, but the specific mechanisms remain unclear. Understanding how microbial metabolites affect epigenetic landscapes in hormone-producing tissues is critical for explaining age-related decline and developing interventions (Borrego-Ruiz and Borrego, 2024; Chatterjee et al., 2025; Gyriki et al., 2025; Ling et al., 2020; Ye et al., 2024). A central question is unresolved: Are age-related changes in the gut microbiota a cause or a consequence of decline? The relationship is bidirectional. The aging host, through changes in hormones, metabolism, and immunity, shapes the microbial ecosystem (Kopalli et al., 2026; Shi et al., 2024). It is also unclear how organs such as the hypothalamus, pancreas, liver, adipose tissue, adrenal glands, and reproductive support cells integrate microbial signals and interact with one another during aging. The reproductive system, often studied for fertility, may be a sensitive indicator of overall metabolic and hormonal stress (Shin et al., 2021; Anway and Skinner, 2008; Vaiserman et al., 2017). Disruption in these networks leads to widespread endocrine dysfunction, not just isolated organ failure.

Researchers are investigating new ways to modulate the gut microbiota. These include dietary interventions, defined microbial metabolites, and fecal microbiota transplantation. The goal is to influence epigenetic aging. Early animal and human studies show these approaches can change both epigenetic patterns and microbial metabolism (Boehme et al., 2023; Dugan et al., 2023; Ni Lochlainn et al., 2024). This review summarizes evidence on how microbiota-derived signals shape epigenetic regulation in endocrine and reproductive tissues during aging. It also explores if these interactions act as causes, modulators, or responses in age-related decline. Finally, critical research gaps must be addressed before microbiota-based therapies can reliably reduce age-related dysfunction in these systems.

## 2 Methodology: a hypothesis-driven narrative review of gut microbiota-epigenetic interactions in systemic aging

### 2.1 Study rationale and priori conceptual framework

This review is divided into three main sections to create a clear and logical structure. The first section explains how microbiota-derived metabolites, such as short-chain fatty acids, bile acids, tryptophan derivatives, and polyamines, affect chromatin states *via* DNA methylation, histone modifications, and nuclear receptor signaling. The second section uses this framework to examine how these mechanisms relate to endocrine and reproductive aging in specific organs, with a summary table (Table 1) to highlight tissue-specific effects and avoid redundancy. The third section discusses the practical and therapeutic implications, including fecal microbiota transplantation, dietary changes, and postbiotic approaches, and also considers current challenges and future research needs.

**TABLE 1:** Evidence hierarchy: we classify studies into four tiers based on the directness of epigenetic measurement and the complexity of the biological system.

Evidence tier	Definition	Experimental systems included	Interpretive strength
Tier 1: Direct chromatin assays *in vivo*	Histone modifications, chromatin accessibility, or DNA methylation measured directly in relevant tissues using ChIP-seq, ATAC-seq, WGBS, or bisulfite sequencing	Animal models (germ-free, colonization, FMT); human tissue biopsies	Strongest – direct mechanistic evidence
Tier 2: Functional *in vivo* evidence without direct chromatin assays	Gene expression changes, metabolic outcomes, or signaling pathway alterations in response to microbiota manipulation; chromatin changes inferred but not measured	Animal models with RNA-seq, metabolomics, or protein assays; pharmacological inhibition of epigenetic enzymes	Intermediate – mechanistically plausible but indirect
Tier 3: Cell culture or *ex vivo* systems	Isolated cell types exposed to defined metabolites; direct chromatin assays often possible, but physiological context lost	Primary cells or cell lines treated with SCFAs, bile acids, indoles, or polyamines	Mechanistic but reductionist – loses physiological context
Tier 4: Human observational studies	Associations between microbiome composition, circulating metabolites, and epigenetic aging markers (e.g., DNA methylation clocks); no direct chromatin measurements in target tissues	Human cohort studies with fecal metagenomics, plasma metabolomics, and blood-based methylation arrays	Correlative only; cannot infer causation

Two main hypotheses shaped this review from the start. First, we suggest that microbiota-derived metabolites act as important links between the body’s metabolic state and chromatin regulation, which affects gene expression and hormone function as we age. Second, the relationship goes both ways: as people age, changes in hormones, metabolism, and the immune system (such as lower sex hormones, insulin resistance, and immune aging) alter the gut environment, which, in turn, affects the microbiome. This shows a two-way connection.

We bring together current research on how microbiota-derived metabolites, such as short-chain fatty acids, bile acids, tryptophan derivatives, and polyamines, affect the body’s epigenetic systems and influence changes in endocrine and reproductive networks. Studies in cells, animals, and humans show that these metabolites interact with DNA methylation, histone modifications, and chromatin structure, thereby shaping gene expression related to metabolism, inflammation, and hormone function. This review also examines new treatment strategies, such as dietary changes, postbiotics, and microbiota targeting, while noting the current limitations of these approaches in achieving lasting epigenetic changes. We clearly describe our methods for finding, selecting, and evaluating studies, following best practices for narrative reviews and relevant PRISMA 2020 guidelines.

### 2.2 Literature search strategy and evidence classification

To be transparent and reduce selection bias, we conducted a structured narrative literature review rather than a formal systematic review or meta-analysis. This approach was chosen because microbiota–epigenetic research is interdisciplinary and changes quickly. We searched the following electronic databases for relevant studies: PubMed/MEDLINE, Web of Science Core Collection, Scopus, and Google Scholar for extra citation tracking. The search included publications from January 2000 to February 2026, which is when research on microbiome–epigenome interactions in aging became more focused. The last search update was in February 2026. We used both controlled vocabulary (such as MeSH terms) and free-text keywords in our search strategies, including but not limited to:

“gut microbiota” OR “microbiome” AND “epigenetics” OR “DNA methylation” OR “histone modification” AND “short-chain fatty acids” OR “microbial metabolites” AND “aging” OR “systemic aging” OR “longevity” AND “endocrine regulation” OR “reproductive aging” AND “chromatin remodeling” OR “mTOR signaling”

We used Boolean operators (AND, OR) in our searches. We also manually reviewed the reference lists of included articles to identify additional relevant studies.

### 2.3 Inclusion and exclusion criteria

#### 2.3.1 Inclusion criteria

We selected studies based on their mechanistic relevance, experimental rigor, and translational significance. We prioritized peer-reviewed original research that examined how microbiota-derived metabolites interact with host epigenetic processes in cell systems, animal models, and human studies. When several studies looked at similar mechanisms, we gave preference to those using direct chromatin-level assays or well-controlled experimental designs.

#### 2.3.2 Exclusion criteria

We excluded non-peer-reviewed reports, such as preprints and conference abstracts without data. We also excluded studies that did not provide mechanistic insight into epigenetic regulation or aging biology, or that only described microbiome composition without functional or molecular analysis.

### 2.4 Evidence classification framework

To separate well-supported mechanisms from more speculative or correlative findings, we used a hierarchical evidence classification framework in this manuscript. Mechanisms were labeled as established if they had consistent experimental validation, especially from direct chromatin assays like chromatin immunoprecipitation sequencing (ChIP-seq), assay for transposase-accessible chromatin sequencing (ATAC-seq), or whole-genome bisulfite sequencing (WGBS). For example, SCFA-mediated HDAC inhibition in intestinal epithelium and butyrate-induced H3K27ac at the Foxp3 locus were considered established. Emerging mechanisms were those mainly supported by preclinical models or limited human data, often using indirect measures such as gene expression or metabolite profiling. Examples include bile acid–FXR signaling in liver aging and AhR-dependent chromatin effects in microglia. Hypothesis-generating concepts were based mostly on associative or correlative findings without direct mechanistic validation, such as microbiota-driven DNA methylation changes in human reproductive tissues or transgenerational epigenetic inheritance *via* the gut microbiome. We refer to this framework in the results and discussion sections to help readers distinguish between direct chromatin-level evidence and findings based on physiological inference.

### 2.5 Bias reduction in narrative synthesis

Because the experimental systems and outcomes were so varied, we used a narrative synthesis approach to bring together molecular, metabolic, and systems-level findings that could not be combined quantitatively. To reduce bias, we systematically searched multiple databases, including both supporting and conflicting studies, and balanced foundational work with recent advances. We also used cross-referencing and forward citation tracking to find influential publications. This structured approach helps integrate concepts and keeps the review transparent, both of which are important for a hypothesis-driven review in aging biology.

### 2.6 Evidence hierarchy and limitations in microbiota–epigenetic aging research

A central challenge in this field is the gap between mechanistic insights from reductionist systems and the complexity of human aging. Throughout this review, we distinguish conclusions based on the experimental systems from which they derive. This distinction is critical for readers to assess the strength of evidence for each tissue discussed.

#### 2.6.1 Limitations of each experimental approach

Understanding these limitations is essential for interpreting the evidence presented in this review.

**Tier 1** limitations (Direct chromatin assays *in vivo*): Requires tissue harvesting; rarely possible in humans except for biopsied or surgically resected tissue. Animal models differ from humans in lifespan, immune system, gut physiology, and microbiome composition. Most studies are cross-sectional; longitudinal chromatin data are scarce. Cost and technical expertise limit widespread application

**Tier 2** limitations (Functional *in vivo* without chromatin assays): Changes in gene expression do not always indicate direct epigenetic regulation. These changes could also come from signaling cascades, post-transcriptional regulation, or shifts in metabolism. Metabolic improvements (e.g., insulin sensitivity) after microbiota manipulation could be independent of chromatin changes. Claims of ‘epigenetic effects’ are often just guesses if there are no chromatin assays to confirm them.

**Tier 3** limitations (Cell culture systems): Metabolite concentrations used *in vitro* often exceed physiological levels found *in vivo*. Isolated cells lack tissue context (neural connections, hormonal signals, immune interactions) Non-immortalized primary cells are difficult to obtain from aged human endocrine tissues.

**Tier 4** limitations (Human observational studies): Correlation does not equal causation; reverse causation (aging → dysbiosis) is equally plausible. Blood-based DNA methylation clocks reflect immune cell epigenomes rather than those of endocrine or reproductive tissues. Confounding by diet, medications, comorbidity, and lifestyle is difficult to fully adjust for. Longitudinal studies with repeated sampling are rare.

#### 2.6.2 How evidence levels are indicated in this review

Part I: Mechanistic Insights into Gut microbiota-epigenetic interactions in systemic aging.

## 3 Microbial-derived metabolites and chromatin regulation

As people age, many organs lose function due to age-related disorders (Chatterjee et al., 2025). These problems are linked to changes in gene regulation, including DNA methylation, histone modifications, and non-coding RNA expression. These epigenetic factors control gene activity and influence how aging progresses (Borrego-Ruiz and Borrego, 2024; Strazhesko et al., 2023; Wang et al., 2022; Wu Z. et al., 2024; Izadi et al., 2024). The gut microbiota affects the rate of epigenetic aging (Ye et al., 2024; Xu H. et al., 2024; Huang et al., 2023). A small group of microbiota-derived metabolites (MDMs) connects microbial activity to gene regulation in aging tissues. The main types are short-chain fatty acids (SCFAs), bile acids, tryptophan derivatives (indoles), and polyamines. These metabolites directly affect chromatin by influencing histone acetylation through HDAC inhibition, thereby impacting DNA modifications (Lin et al., 2024)**.** Mendelian randomization studies have found a causal link between certain gut microbiota compositions and faster biological aging, including phenotypic age acceleration *via* DNA methylation (Ye et al., 2024; Huang et al., 2023). Recent studies in humans have developed a ‘gut microbial age’ measure linked to cardiovascular disease risk in metabolically unhealthy older adults (Gut microbial age modulates cardiovascular, 2024). These findings support a connection between gut microbiota and aging, but they do not prove causation. Long-term intervention studies are still needed (Rubas et al., 2025). The next sections in 3.3 describe how each SCFA affects chromatin structure.

### 3.1 DNA methylation regulation by microbiota-derived metabolites

Microbial metabolites have a strong impact on DNA methylation, especially as people age (Borrego-Ruiz and Borrego, 2024; Rubas et al., 2025; Sumi and Kartha, 2022; Shock et al., 2021). This mainly occurs because these metabolites alter the availability of methyl donors, which are regulated by DNA methyltransferases (DNMTs) and metabolic cofactors (Rubas et al., 2025; Leesang et al., 2024) The gut microbiota produces substances such as methylamines, enzymes, vitamins, and short-chain fatty acids (SCFAs), including butyrate, acetate, and propionate, all of which help the host and microbiome communicate (Shock et al., 2021; Stein and Riber, 2023; Durrant et al., 2021; Salvi and Cowles, 2021). SCFAs can influence DNA methylation by changing how cells use energy and process one-carbon metabolism (Stein and Riber, 2023). Gut bacteria make SCFAs by fermenting dietary fiber (Neri-Numa and Pastore, 2020; Modoux et al., 2022). Research shows that SCFAs can alter DNA methylation linked to diabetes risk (Guo et al., 2022a); and varying amounts of butyrate, propionate, and acetate can affect gene expression in human intestinal cells (Grouls et al., 2022). Recent studies from Michael P. Snyder’s lab have shown how microbiota-derived metabolites can directly affect chromatin regulation through unique histone acylations (Nshanian et al., 2024). The researchers used several advanced methods, including LC–MS/MS, ChIP-seq, ATAC-seq, RNA sequencing, and CUT&Tag profiling in both cell and mouse intestinal tissue models (Seligson et al., 2005; Hałasa et al., 2019). They found that SCFA-derived acylations are added to histones in regions of DNA that are actively being transcribed. These changes were associated with increased chromatin accessibility and the activation of genes involved in metabolism, cell growth, and stress responses. This research expands on the traditional view that SCFAs inhibit histone deacetylases, showing that microbial metabolites can also serve as direct substrates for histone acylation.

This process alters chromatin structure by adding specific epigenetic marks. These findings show that microbial metabolic products can directly affect host gene expression through unique histone modifications. However, because most of this research comes from cell and animal studies, it remains unclear how well these results apply to aging and hormone regulation in humans.

Cell and animal studies provide direct evidence (Tier 1) that the microbiota can change histone acetylation, chromatin accessibility, and DNA methylation at specific sites (Pan W-H. et al., 2018; Krautkramer et al., 2017); For example, in zebrafish, transplanting young fecal microbiota into older fish rejuvenates the reproductive endocrine system and reduces toxicity, suggesting a strong epigenetic effect (Tang et al., 2022). In germ-free mice, there is less Tlr4 promoter methylation in colonic epithelial cells, which is associated with lower gene expression and reduced response to lipopolysaccharides. This supports the idea that the microbiota helps shape the host epigenome during development (Takahashi et al., 2009). These methylation changes are a downstream response to larger metabolic shifts, which will be discussed later. Surface colonocytes use butyrate for energy, and when stem cells are exposed to butyrate, they show higher histone acetylation but have reduced ability to proliferate or repair (Donohoe et al., 2011).

Changes in the gut microbiome also affect one-carbon metabolism in the body by influencing the folate and methionine cycles, which are important for cellular methylation. These cycles help produce S-adenosylmethionine (SAM), the main methyl donor needed for DNA methyltransferase (DNMT) to methylate cytosine (Leesang et al., 2024; van Vliet et al., 2024; Lionaki et al., 2022). The methionine cycle creates SAM, and both diet and gut microbes provide the nutrients needed for enough methyl groups to support epigenetic regulation (Anderson et al., 2012). DNMT3A and DNMT3B are *de novo* methyltransferases that set up new DNA methylation patterns during development and when the environment changes. DNMT1, on the other hand, keeps existing methylation marks during DNA replication, which helps maintain epigenetic stability as cells divide (Stein and Riber, 2023). If microbial metabolism is disrupted, it can change the levels of methyl donors and intermediates like SAM and S-adenosylhomocysteine (SAH), which in turn can affect DNMT activity and DNA methylation patterns.

**FIGURE 2:**
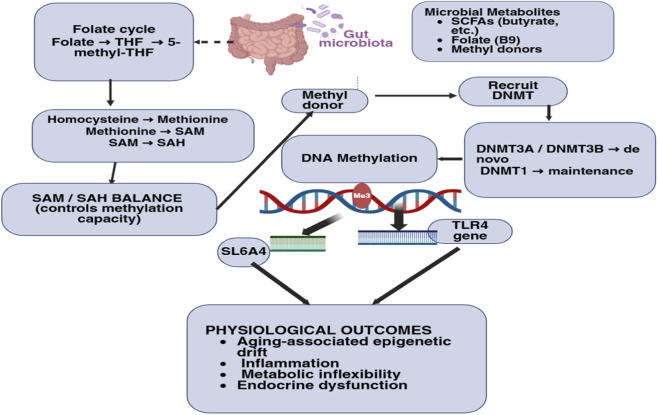
How gut microbiota influence host DNA methylation through methyl-donor metabolism and DNMT recruitment. The diagram shows that microbial metabolites (SCFAs, folate, methyl donors) feed into the folate cycle (folate → THF → 5-methyl-THF) and the methionine cycle (homocysteine → methionine → SAM → SAH). The SAM/SAH balance controls the cell’s overall methylation capacity. Elevated SAM recruits DNMTs: DNMT3A/3B for *de novo* methylation and DNMT1 for maintenance methylation. This leads to locus-specific DNA methylation changes, exemplified by the TLR4 gene. The resulting physiological outcomes include aging-associated epigenetic drift, inflammation, metabolic inflexibility, and endocrine dysfunction.

These changes in DNA methylation, driven by metabolism, are closely connected to important aspects of aging, such as chronic inflammation, reduced metabolic flexibility, and changes in gene expression (Vaccaro and Naser, 2022). When there are fewer methyl donors or imbalances in one-carbon metabolism, it can cause overall hypomethylation and specific areas of hypermethylation, which are often seen in aging and age-related diseases as indicated in intestinal epithelial cells (Pan W-H. et al., 2018; Krautkramer et al., 2017). Because of this, the way the microbiota controls the methionine cycle is a key link between environmental and dietary factors and how they influence the host epigenome over time. Figure 2 shows these interactions in a diagram

### 3.2 Active DNA demethylation and TET enzyme regulation

Metabolites produced by the microbiota can influence active DNA demethylation by regulating ten-eleven translocation (TET) enzymes (Rubas et al., 2025; Leesang et al., 2024). TET proteins need iron and α-ketoglutarate to convert 5-methylcytosine (5mC) into 5-hydroxymethylcytosine (5hmC), 5-formylcytosine (5 fC), and 5-carboxylcytosine (5caC), which helps maintain methylation turnover and flexible gene expression (López-Moyado et al., 2024; Ma L. et al., 2022). The activity of TET enzymes depends on the cell’s metabolic state, including levels of α-ketoglutarate, oxygen, and iron. Microbiota-driven pathways can change TCA cycle intermediates and affect redox balance (Leesang et al., 2024). If these pathways are disrupted, for example by the oncometabolite 2-hydroxyglutarate, TET-mediated demethylation is impaired, and DNA methylation increases (Leesang et al., 2024).

**FIGURE 3:**
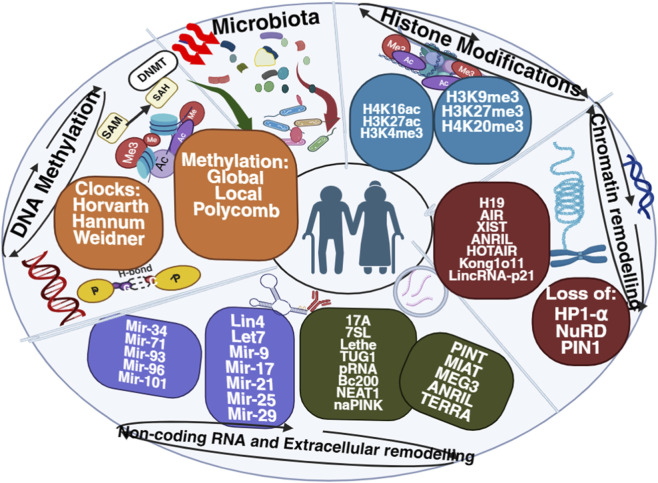
Interconnected epigenetic mechanisms influenced by metabolic signals across the lifespan. The diagram illustrates three interconnected layers of epigenetic regulation affected by microbiota: DNA methylation, histone modifications, and non-coding RNAs. DNA methylation clocks (Horvath, Hannum, Weidner) track biological age. Microbiota influence global, local, and Polycomb-group methylation, as well as DNMT activity and SAM/SAH balance. Age-associated alterations include loss of chromatin proteins (HP1-α, NuRD, PIN1), redistribution of repressive histone marks (H3K9me3, H3K27me3, H4K20me3) and activating marks, converging to influence cellular identity, tissue maintenance, and organismal aging trajectories. Non-coding RNAs (lin4, let-7, miR-9, -17, -21, -25, -29) and long non-coding RNAs (H19, AIR, XIST, ANRIL, HOTAIR, *etc.*) mediate extracellular vesicle-dependent chromatin remodeling. Age-associated alterations include redistribution of repressive marks (H3K9me3, H3K27me3, H4K20me3) and activating marks, converging to influence cellular identity, tissue maintenance, and organismal aging trajectories.

DNA hydroxymethylation helps prevent excessive variation in gene expression as we age, suggesting that active demethylation stabilizes age-related epigenetic changes (Occean et al., 2024). Changes in the microbiome that come with age may reduce the metabolic support needed for balanced methylation and demethylation, leading to increased inflammation and unstable gene expression (Sun et al., 2020; Sun et al., 2022; Krepelova et al., 2025). Studies in humans have found that the makeup of the microbiome is linked to faster epigenetic aging. Experiments show that exposing organisms to different microbiota changes CpG island methylation compared to germ-free conditions (Sun et al., 2020; Sun et al., 2022). Interactions between the host and microbiota contribute to epigenetic drift, the accumulation of random methylation changes now recognized as a key feature of aging (Fan X. et al., 2025). Current evidence shows that changes in methylation linked to the microbiota depend on the context and mostly come from preclinical or correlative human studies (Rubas et al., 2025; Shock et al., 2021). Microbial metabolites help regulate epigenetic flexibility as part of larger genetic, environmental, and metabolic systems (Galow and Peleg, 2022). See Figure 3.

### 3.3 Metabolic control of histone acetylation

Histone acetylation and methylation serve as quick, reversible connections between metabolic signals and gene expression (Borrego-Ruiz and Borrego, 2024; Izadi et al., 2024; de Lima Camillo et al., 2025), especially in microbiota-host communication during aging (Zhou et al., 2021; Galow and Peleg, 2022; Gates et al., 2024). Acetylation removes the positive charge from histones, loosening their grip on DNA and creating an open chromatin state (euchromatin) that helps transcription (Ali et al., 2022; Xie et al., 2023). Methylation of histones can either turn genes on or off, depending on the specific site and methylation level (de Lima Camillo et al., 2025). Enzymes that modify chromatin need metabolic cofactors, so changes in microbial metabolism can affect histone modifications by changing host metabolism. Histone acetyltransferases (HATs) and HDACs depend on acetyl-CoA and NAD^+^ (Thomas and Denu, 2021; Lund et al., 2022), which directly connect cellular metabolism to gene expression (Wu et al., 2020).

#### 3.3.1 SCFAs as HDAC inhibitors

Among microbial metabolites, short-chain fatty acids (SCFAs) like acetate, propionate, and butyrate are most studied for their effects on chromatin (Lee et al., 2024; Mann et al., 2024; Martin-Gallausiaux et al., 2020; Wang et al., 2024c). SCFAs are produced when bacteria ferment dietary fiber (Fock and Parnova, 2023; Liu M. et al., 2024; Visekruna and Luu, 2021). Butyrate and propionate block Class I and II HDACs (Mann et al., 2024; Martin-Gallausiaux et al., 2020; Zhang D. et al., 2023). This prevents the removal of acetyl groups from histones, increases histone acetylation, and allows easier transcription factor access to gene promoters (Lund et al., 2022; Shi et al., 2021). Strong experimental evidence shows SCFAs mainly change histone acetylation in intestinal and immune tissues (Zuo et al., 2024; Li Y. et al., 2024; Eshleman et al., 2024). Inhibiting HDACs does not simply activate all genes; effects depend on local transcription factors, metabolic state, and chromatin structure (Farhadipour et al., 2023). The most consistent mechanism is SCFA-dependent HDAC inhibition, seen directly in intestinal and immune systems and, more recently, in metabolic organs. For example, this study uses direct ChIP-seq evidence for SCFA-responsive histone acylations in human intestinal cells (Kabir et al., 2025). Another proposed study finds that two common SCFAs, butyrate and pentanoate, influence T cell differentiation *in vitro* (McBride et al., 2023). In the intestinal epithelium, SCFA-driven histone acetylation is demonstrated through chromatin assays, including ChIP-seq, ATAC-seq, and mass spectrometry-based histone modification profiling (see Table 2.5.1 Tier 1).

Whole-genome bisulfite sequencing (WGBS) and ChIP-seq analyses in germ-free *versus* conventionalized mice show that the microbiota influences DNA methylation and H3K4me3/H3K27ac levels in colonic epithelial cells (Ansari et al., 2020). Also, butyrate treatment induces widespread increases in H3K27ac and H3K9ac in mouse colonic epithelium and human colon cancer cell lines (Zuo et al., 2024; Li Y. et al., 2024; Eshleman et al., 2024). ChIP-seq demonstrated that histone butyrylation (H3K27bu) is detectable in human colon samples and is responsive to SCFA levels (Kabir et al., 2025). Microbiota-dependent histone acylations (acetylation, butyrylation, propionylation) are associated with active gene regulatory elements in intestinal epithelial cells (Sumi and Kartha, 2022; Kabir et al., 2025; Kang et al., 2023).

In Colorectal cancer cells and *in vitro* systems: CUT&Tag and ChIP-seq in human colorectal cancer cells and normal colon epithelial cells mapped genome-wide locations of four short-chain acyl histone marks revealed that propionate and butyrate directly modify histones as unique acyl marks (H3K18pr, H3K18bu, H4K12pr, H4K12bu), correlating with open chromatin and gene expression (Nshanian et al., 2024). SCFAs affect acetyl-CoA supply (Saiman et al., 2021), which is synthesized from Acetate conversion to acetyl-CoA by ACSS1 and ACSS2, providing substrate for HATs (Thomas and Denu, 2021; Kalkan et al., 2025), directly linking microbial carbon flow to open chromatin (Lund et al., 2022). Propionate is used in mitochondrial metabolism as succinyl-CoA (Gates et al., 2024). Butyrate provides energy to colon cells and supplies carbon for the acetyl-CoA pathway (Liu M. et al., 2024; Mohamed et al., 2023). Acetate increases histone H3 acetylation, which activates the lipogenic genes ACACA and FASN by raising H3K9, H3K27, and H3K56 acetylation at their promoters. This process, controlled by acetyl-CoA synthetases ACSS1 and ACSS2, boosts new lipid production and supports acetate’s role as a fatty acid precursor (Gao et al., 2016). Stable isotope tracing shows that microbial butyrate contributes to histone acetylation in intestinal cells (Gates et al., 2024; Lund et al., 2022). SCFAs also protect the heart through DNA methylation pathways. In people with type 2 diabetes, lower levels of Faecalibacterium prausnitzii (an important butyrate producer) are associated with greater methylation at free fatty acid receptor promoter CpG sites (Chleilat et al., 2021).

Age-related decline in immune regulation is linked to a decrease in butyrate-producing bacteria such as Faecalibacterium and Roseburia as people get older (Protachevicz et al., 2026; Best et al., 2025), lowering intestinal and systemic SCFA levels. In people over 80, butyrate levels fall sharply (Liu H. et al., 2018; Hodgkinson et al., 2023; Van-Wehle and Vital, 2024), which is associated with less histone acetylation, tighter chromatin, more inflammatory gene activity, and weaker mitochondrial function (Deng et al., 2025). These changes together affect gene regulation in endocrine, immune, and metabolic tissues (Izadi et al., 2024; Galow and Peleg, 2022; de Lima Camillo et al., 2025). In immune cells Direct ChIP-seq evidence exists for SCFA effects on immune cell chromatin: Butyrate increases H3K27ac at the Foxp3 locus in T cells, promoting regulatory T cell differentiation (Kespohl et al., 2017). SCFAs alter chromatin accessibility in B cells *via* HDAC inhibition, affecting antibody responses. Studies in mouse T cells show that butyrate regulates histone acetylation and can inhibit CRC tumor growth by increasing cell death, inducing cell cycle arrest, and reducing inflammation (Saldanha et al., 2014; Donohoe et al., 2014). These results highlight butyrate’s protective role in the colon and its potential to prevent or treat CRC. ChIP-qPCR has measured histone markers on genes that suppress inflammation. CRC tumors from mice on a high-fat diet had higher H3ac levels at FAS promoters, showing butyrate’s effect on cell death and its role as an HDAC inhibitor (Donohoe et al., 2014). Macrophages and dendritic cells detect SCFAs through G protein-coupled receptors, which increases global H3 acetylation and boosts anti-inflammatory cytokine expression and Treg cell activity (Björkegren and Lusis, 2022). ChIP-seq and ATAC-seq in B cells showed SCFA-dependent chromatin accessibility changes affecting antibody responses (Grandi et al., 2022). In mouse hematopoietic stem cells (Tier 1 – Direct): transplanting fecal microbiota from young to old mice can reverse age-related changes in histone marks (H3K27ac, H3K4me1, H3K4me3), as shown by ChIP-seq (Zeng et al., 2023).

#### 3.3.2 Nuclear receptor and GPCR signaling

**FIGURE 4:**
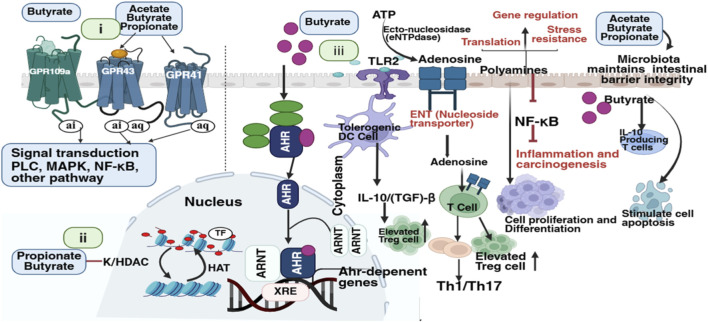
Microbiota-immune-metabolite signaling pathways. The diagram shows three primary routes by which SCFAs (butyrate, acetate, propionate) act on target cells: (i) SCFAs bind to GPCRs (GPR109A, GPR43, GPR41) on the cell membrane, leading to signal transduction through PLC, MAPK, on intestinal epithelial and immune cells, leading to inhibition of NF-κB signaling, reduced pro-inflammatory cytokine production and other pathways. (ii) Propionate and butyrate enter the cell, move to the nucleus, inhibit HDAC, and activate HAT, resulting in increased histone acetylation and gene regulation. (iii) SCFAs enter the cell with the help of AhR, then translocate to the nucleus where AhR and ARNT bind DNA (XRE) to regulate gene expression. Tryptophan metabolites (IAA, IPA, IAld) also activate AhR signalling. Microbiota regulate extracellular ATP metabolism *via* ecto-nucleosidase (eNTPdase), generating adenosine, which promotes IL-10 and TGF-β production, leading to elevated Treg cells and suppression of Th1/Th17 responses. Butyrate further enhances Treg differentiation and supports epithelial integrity. The bottom of the diagram indicates the evidence translation spectrum: *in vitro* models → animal models → human observational studies → clinical intervention evidence.

Besides directly inhibiting HDACs, SCFAs also activate GPCRs such as FFAR2 (GPR43), FFAR3 (GPR41), and GPR109A (HCAR2), which are found on immune and enteroendocrine cells (Overby and Ferguson, 2021; Liu et al., 2021) When activated, these receptors trigger cAMP, MAPK, and PI3K-Akt pathways (Mann et al., 2024; Overby and Ferguson, 2021), affecting chromatin accessibility by altering transcription factor phosphorylation and cofactor recruitment, thereby limiting NF-κB-dependent inflammatory transcription (Shi et al., 2021; Sanchez et al., 2020). Most of these detailed mechanisms come from experimental or preclinical studies, while human research mainly shows associations (Best et al., 2025; Guo et al., 2023). Microbial metabolites help regulate chromatin flexibility, adjusting transcriptional responses within broader genetic, environmental, and metabolic settings (Shock et al., 2021; Li D. et al., 2022). See Figure 4.

### 3.4 Nutrient-dependent signals in mTOR regulation

The mechanistic Target of Rapamycin (mTOR) pathway integrates nutrient availability, cellular metabolism, and transcriptional regulation during aging (Wang Y. et al., 2024; Gao and Tian, 2023; Mota-Martorell et al., 2020; Cochemé and Gil, 2024; Zhao et al., 2024). The gut microbiota influences nutrient sensing and substrate metabolism, thereby connecting microbial signals to chromatin regulation *via* mTOR (Missong et al., 2024; Ferenc et al., 2024). Metabolites produced by the microbiota indirectly modulate transcriptional stability during aging by coordinating metabolic and chromatin networks (Missong et al., 2024; Tian et al., 2024).

#### 3.4.1 AMPK activation

Short-chain fatty acids (SCFAs) activate AMP-activated protein kinase (AMPK), which opposes mTOR complex activity. AMPK acts as a cellular energy sensor, becoming active under low ATP conditions and inhibiting mTORC1 signaling and anabolic pathways (Wang Y. et al., 2024; Gao and Tian, 2023; Mota-Martorell et al., 2020; Cochemé and Gil, 2024; Zhao et al., 2024). Activation of AMPK promotes healthy aging and metabolic homeostasis. For example, AMPK enhances miR-708 maturation, which reduces DAB2 expression and mTORC1 activity (Yi et al., 2021).

#### 3.4.2 mTOR activation

Microbe-induced changes in amino acid availability, bile acid signaling, and insulin sensitivity can result in context-dependent mTOR activation (Gao and Tian, 2023; Fan et al., 2022), affecting transcriptional programs controlling protein synthesis, autophagy, mitochondrial function, and cellular aging (Chakraborty et al., 2024; Martin et al., 2021; Liang et al., 2020). mTORC1 activation increases H3K27me3, thereby affecting cell growth (Kim et al., 2024). Nuclear mTOR regulates transcription factors, epigenetic changes, and chromatin remodeling (Ge et al., 2022; Skjærven et al., 2023). Many chromatin modifiers require acetyl-CoA, α-ketoglutarate, NAD^+^, or ATP as cofactors (Klotz and Carlberg, 2023), ensuring epigenetic state and metabolic condition remain connected (Fan et al., 2026). This relationship is particularly evident in cancer cells, where metabolic reprogramming and epigenetic dysregulation frequently co-occur (Ge et al., 2022).

#### 3.4.3 Aging and inflammation

**FIGURE 5:**
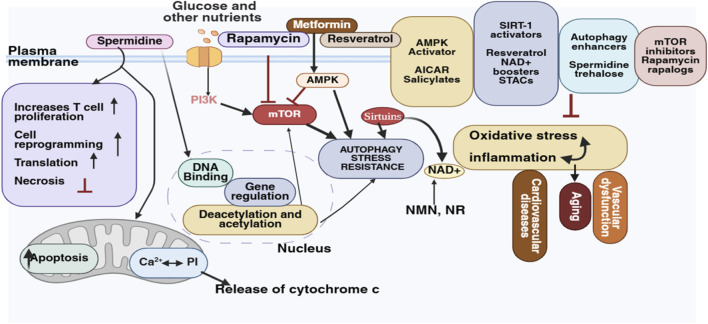
The central role of mTOR in integrating nutrient signals that influence aging and longevity. Schematic representation of the mTOR signaling pathway integrating nutrient, pharmacological, and microbiota-derived signals in the regulation of cellular metabolism, autophagy, and aging. Nutrient availability (e.g., glucose and amino acids) activates upstream pathways such as PI3K/AKT, leading to activation of mTOR complex 1 (mTORC1), which promotes protein synthesis and cell growth while inhibiting Autophagy. Energy stress activates AMPK, which inhibits mTORC1 and promotes autophagy and metabolic adaptation. Pharmacological agents, including Metformin and Rapamycin, suppress mTOR signaling directly or *via* AMPK activation, whereas Resveratrol and NAD^+^ precursors enhance sirtuin activity, further modulating metabolic and stress-response pathways. Microbiota-derived metabolites, such as short-chain fatty acids and polyamines (e.g., spermidine), influence host signaling by activating AMPK and sirtuin pathways, thereby inhibiting mTORC1 and enhancing autophagy. Collectively, these interactions reduce oxidative stress and inflammation, improve cellular stress resistance, and contribute to the regulation of aging and longevity. Downstream effects include modulation of oxidative stress, inflammation, and age-associated cardiovascular and metabolic dysfunction.

Aging is characterized by increased mTORC1 activity, reduced AMPK activity, and persistent low-grade inflammation, commonly referred to as “inflammaging (Blasiak et al., 2020). Other aging hallmarks include a shift toward glycolysis, reduced mitochondrial function, and activated inflammatory pathways (Fan J. et al., 2025). Targeting mTOR, boosting AMPK, or restoring NAD^+^ levels may slow age-related decline (Guo J. et al., 2022; Gao et al., 2023; Li et al., 2021). However, most evidence supporting these interventions is derived from experimental models, and the influence of the microbiota on mTOR regulation in humans remains an emerging research area (Fan and Xu, 2026; Donati Zeppa et al., 2022; Li and Roy, 2023). See Figure 5.

### 3.5 Major classes of microbial-derived metabolite signaling

#### 3.5.1 Bile acid signaling

Bile acids are steroid molecules made from cholesterol in the liver. Gut bacteria modify them through processes like deconjugation and dehydroxylation (bile salt hydrolase, 7α-dehydroxylase), creating secondary bile acids such as deoxycholic acid (DCA) and lithocholic acid (LCA) (Guzior and Quinn, 2021; Larabi et al., 2023). These secondary bile acids act as endocrine ligands and can directly affect chromatin by interacting with nuclear receptors (FXR, PXR) and the membrane receptor TGR5 (GPBAR1), which help regulate signaling by altering bile acid levels (Wahlström et al., 2016). In obese mice fed a high-fat diet, changes in bile acid profiles were associated with lower GLP-1 levels, but treatment with UDCA (ursodeoxycholic acid) improved these metabolic abnormalities (He et al., 2026). Bile acids help maintain the intestinal immune barrier by regulating effector T cells. They also influence host immunity by affecting the balance between Th17 and regulatory T (Treg) cells, as well as controlling intestinal RORγ+ Treg cells (He et al., 2026).

##### 3.5.1.1 FXR signaling

When activated, FXR pairs with RXR and binds to response elements at genes involved in metabolism and inflammation (KANAYA et al., 2004). Under healthy conditions, FXR recruits coactivators (SRC-1, p300), which increase histone acetylation at genes that regulate bile acid balance, fat breakdown, and insulin sensitivity (Caron et al., 2006; Eloranta and Kullak-Ublick, 2008). As people age and microbial bile acid patterns change, FXR signaling leads to reduced enhancer acetylation and weaker repression of inflammatory genes, disrupting enhancer structure in liver and intestinal cells.

##### 3.5.1.2 TGR5 signaling

Secondary bile acids activate TGR5, which leads to CREB phosphorylation and brings HATs to metabolic enhancers (Agus et al., 2021; Koh et al., 2016). In enteroendocrine cells, this augments GLP-1 secretion from L cells in the intestine and this secretion can also protect against insulin resistance (Saad et al., 2016); in macrophages and microglia, it attenuates NF-κB-dependent chromatin accessibility (Tian et al., 2022). This means that gut microbes, by altering bile acid composition, create a dual-receptor system that helps stabilize enhancers. When aging alters gut bacteria, the balance between primary and secondary bile acids shifts, making it harder to repress inflammatory chromatin and activate metabolic enhancers. Modifying the gut microbiota in mice can reverse age-related inflammation and bile acid imbalance (Ma et al., 2020).

#### 3.5.2 Tryptophan–AhR signaling

Gut microbes break down tryptophan into indole derivatives (IPA, IAA, IAld, tryptamine) that affect gene regulation through the aryl hydrocarbon receptor (AhR). Different indole ligands change the shape of the receptor, which influences how it pairs with ARNT, how long it stays in the nucleus, and how it recruits coactivators (p300/CBP, SRC) (Gargaro et al., 2021), These ligands also affect the recruitment of histone modifiers (KDM6B, EZH2) and interactions with the SWI/SNF chromatin remodeling complex (Gutiérrez-Vázquez and Quintana, 2018; Roager and Licht, 2018; Janiga-MacNelly et al., 2025).

##### 3.5.2.1 Protective ligands (e.g., IPA)

Certain gut-derived molecules (like indole-3-propionic acid, IPA) act as protective signals. They bind to a receptor called AhR, which then partners with a protein called p300. This partnership adds an acetyl group to histone H3 at a specific location (H3K27ac) near genes that are important for gut defense (Gargaro et al., 2021; Barreira-Silva et al., 2025). These genes include: IL-22 – a cytokine that helps repair the gut lining and fight bacteria; Antimicrobial peptides–molecules that kill harmful microbes; Tight junction genes, proteins that seal the gaps between gut lining cells. The result is a stronger gut barrier and a calmer immune system (immune tolerance). So, protective ligands help keep the gut wall intact and prevent unnecessary inflammation.

##### 3.5.2.2 Pro-inflammatory ligands (kynurenine pathway activation of AhR)

These ligands keep AhR in the nucleus for a longer time, bring in repressive methyltransferase complexes, increase H3K9me2/3 marks, lower barrier enhancer activity, and allow inflammatory genes to be activated (Kurita et al., 2014). AhR also works with NF-κB and STAT3 at shared enhancers. As people age, changes in gut microbes, leading to increased availability of indole ligands, result in less acetylation at barrier enhancers while inflammatory gene activity stays the same or increases (Chen et al., 2026). Preclinical models and multi-omics profiling establish the causal protective role of SCFAs in AS progression by mapping vascular epigenetic remodeling. Butyrate and propionate promote Treg generation both by enhancing Foxp3 acetylation through HDAC inhibition and by serving as acyl-CoA donors for histone acetyltransferases (Arpaia et al., 2013; Thomas and Denu, 2021).

#### 3.5.3 One-carbon metabolism and polyamines

One-carbon metabolism involves the folate cycle, methionine cycle, and transsulfuration pathway to maintain cellular SAM levels. SAM is the main methyl donor for DNA, histone, and RNA methylation (Locasale, 2013). The SAM/SAH ratio acts as a metabolic rheostat: high SAM/SAH supports methylation; low SAM/SAH inhibits it (Leesang et al., 2024). Disruption leads to specific methylation errors that accumulate with age (Islam et al., 2022; Friso et al., 2017). Nutrients like folate, vitamin B6, and B12 are important for one-carbon metabolism and help regulate DNA methylation. If these nutrients are lacking, homocysteine levels rise, which can lead to insufficient methyl donors, reduced DNA methylation, problems with blood vessels, and a faster progression of atherosclerosis (Ma et al., 2017) SCFAs do not supply methyl groups directly, instead, they act as metabolic regulators by controlling carbon flux, redox state, and cofactor levels, thereby indirectly affecting one-carbon metabolism and methylation capacity. SCFAs influence the movement of SAM and SAH, acetyl-CoA levels, TCA cycle intermediates, and the NAD^+^/NADH balance, and they regulate enzymes like S-adenosylhomocysteine hydrolase (Petrova et al., 2023; Ducker and Rabinowitz, 2017). Methionine adenosyltransferase (MAT2) is regulated by mitochondrial redox state *via* propionate-derived succinyl-CoA (Saiman et al., 2021; Choi and Friso, 2023).

##### 3.5.3.1 Polyamines

Polyamines such as spermidine and spermine are produced by both the host and microbes. They are positively charged at normal body pH and help stabilize nucleosomal DNA, which reduces random chromatin opening (Eisenberg et al., 2016). Making polyamines requires decarboxylated SAM, so polyamine production is linked to methylation capacity. As people age, a decline in microbial polyamine production disrupts this balance, leading to hypomethylation (Ciccarone et al., 2018). Spermidine also supports autophagy by inhibiting HATs and increasing the accessibility of autophagy-related genes. Together, SCFAs, secondary bile acids, indole derivatives, and polyamines constitute a microbiota-derived epigenetic metabolome that integrates metabolic flux, nuclear receptor signaling, transcription factor binding, and methyl donor balance (Wang et al., 2023).

After explaining how microbiota-derived metabolites regulate chromatin states through HDAC inhibition, nuclear receptor signaling, AhR activation, and methyl donor balance, **Part II** will apply this framework to specific endocrine and reproductive organs. The next section uses Table 2 to summarize the shared mechanisms across tissues, helping avoid redundant descriptions and highlighting how epigenetic aging appears differently in each organ.

**TABLE 2:** To help readers rapidly assess the strength of evidence for each claim, we use the following indicators in figures and in text where appropriate.

Symbol	Meaning
■■■■■	Tier 1: Direct chromatin assays *in vivo* (animal or human tissue)
■■■■□	Tier 2: Functional *in vivo* evidence without direct chromatin assays
■■■□□	Tier 3: Cell culture or *ex vivo* systems
■■□□□	Tier 4: Human observational studies (associations only)
■□□□□	Clinical intervention evidence (rare; mostly Tier 4 unless biopsy-proven)

Example **from** Figure 2 **in this review:**

Summary of evidence supporting these mechanisms: ■■■■□ *In vitro* chromatin assays; ■■■■■ Animal models; ■■□□□ Human observational methylation studies; ■□□□□ Clinical intervention evidence”.

In the text, when a mechanistic statement is made without an explicit evidence indicator, readers should assume the evidence is **Tier 2 or Tier 3** unless otherwise noted. Statements explicitly labeled as “established” require Tier 1 evidence in at least two independent studies. Statements labeled as “emerging” derive from Tier 2 or Tier 3 evidence. Statements labelled as “hypothesis-generating” derive from Tier 4 or limited Tier 2 evidence. This framework does not diminish the value of lower-tier evidence. Hypothesis-generating and emerging mechanisms (Tiers 2–4) are essential for guiding future research. However, distinguishing these from established mechanisms (Tier 1) prevents premature conclusions and focuses translational efforts on the most robust findings.

Part II: Organ-Specific Endocrine and Reproductive Effects”.

## 4 Organ-specific endocrine and reproductive aging

The chromatin-regulatory pathways discussed in Section 3 provide a shared molecular framework for how microbiota-derived metabolites affect aging in different tissues. However, the same metabolite can have different effects depending on the organ. These tissue-specific outcomes are due to differences in metabolite transporter expression (such as MCT1/2 in the hypothalamus *versus* OATP transporters in the liver), nuclear receptor types (like FXR in the liver *versus* AhR in microglia), baseline chromatin landscapes, and local metabolic needs. To avoid repeating the same mechanisms across tissues, Table 2 summarizes the main epigenetic mechanisms shared across endocrine and reproductive organs, and each subsection then focuses on organ-specific features. Additional details on microbiota-derived metabolites and direct experimental evidence are provided in Tables 3–5, respectively. Most evidence from metabolically active organs comes from gene expression studies, metabolic profiling, or changes in signaling pathways, rather than direct measurements of chromatin accessibility (Li X. et al., 2024; Zhang et al., 2023b; Shon et al., 2024; Wang et al., 2019). These tissue-specific outcomes are due to differences in transporter expression, nuclear receptor types, and chromatin landscapes, not because the metabolites act differently. This review introduces the MDME axis framework and systematically examines the evidence for MDM-mediated epigenetic regulation in different tissues (Lei et al., 2025; Guo et al., 2025). When there is direct chromatin evidence (e.g., ChIP-seq, ATAC-seq, or WGBS), it is noted. If not, the mechanisms discussed are still emerging or are based on hypotheses

**TABLE 3:** List of the experimental systems that provide direct chromatin-level evidence (Tier 1: ChIP-seq, ATAC-seq, WGBS, or mass spectrometry-based histone profiling) supporting this claim.

Experimental system	Technique	Key finding	Citation
Mouse colonic epithelium (germ-free vs. conventionalized)	WGBS, ChIP-seq	Microbiota drives DNA methylation and H3K4me3/H3K27ac changes at metabolic and immune loci	Pan et al., *Genome Med* 2018
Mouse colonic epithelium (butyrate/tributyrin)	ChIP-seq (H3K27ac, H3K27bu, H3K9ac)	Histone butyrylation is microbiota-dependent and associated with active regulatory elements	Gates et al., *Nat Metab* 2024
Mouse T cells (butyrate)	ChIP-seq (H3K27ac)	Butyrate increases H3K27ac at *Foxp3* locus, promoting Treg differentiation	Kespohl et al., *Front Immunol* 2017
Mouse B cells (SCFAs)	ChIP-seq, ATAC-seq	SCFA-dependent chromatin accessibility changes affecting antibody responses	Sanchez et al., *Nat Commun* 2020
Mouse hematopoietic stem cells (heterochronic FMT)	ChIP-seq (H3K27ac, H3K4me1, H3K4me3)	Young FMT reverses age-related histone mark changes in aged HSCs	Zeng et al., *Blood* 2023
Human colorectal cancer cells (butyrate/propionate)	CUT&Tag, ChIP-seq	Propionate and butyrate directly modify histones as unique acyl marks (H3K18pr, H3K18bu, *etc.*)	Nshanian et al., *Nat Metab* 2025
Mouse pancreatic islets (butyrate)	ChIP-seq (H3K27ac)	Butyrate increases H3K27ac at promoter regions of hormone secretion genes	Pedersen et al., *FEBS J* 2024
Mouse brain (secondary bile acid GDCA)	ATAC-seq	Microbiome-derived GDCA regulates chromatin accessibility in the brain	Chakraborty et al., *bioRxiv* 2025

In this review, without explicit reference to a specific tissue, readers should understand that the strongest direct evidence comes from intestinal epithelium, immune cells, and hematopoietic stem cells. Direct chromatin evidence in endocrine organs (hypothalamus, pancreas, liver, adipose, gonads) remains limited or absent, and claims are inferred from gene expression or metabolic changes (Tiers 2–3).

**TABLE 4:** Microbiota-derived metabolites: core epigenetic mechanisms and organ-specific effects.

Metabolite class	Core epigenetic mechanism	Primary mark	Key organ-specific outcomes	Evidence level
**Short-chain fatty acids** (butyrate, propionate, acetate)	Class I/II HDAC inhibition → increased histone acetylation	H3K9ac, H3K27ac	**Hypothalamus:** *POMC, BDNF, IRS2*; **Pancreas:** insulin, *PDX1, MAFA*; **Liver:** *PGC-1α, FOXO1, HNF4α*; **Adipose:** *ADIPOQ, GLUT4*; **Gonads:** steroidogenic enzymes	**Established** (gut/immune chromatin); **Emerging** (endocrine organs)
**Secondary bile acids** (DCA, LCA, UDCA)	FXR/TGR5/PXR signaling → coactivator (SRC-1/p300) or HDAC recruitment	H3K27ac (activation) or H3K9me3 (repression)	**Liver:** bile acid and lipid homeostasis; **Intestine:** *FGF19*; **Macrophages:** NF-κB suppression	**Emerging** (cell and animal models)
**Tryptophan indoles** (IPA, IAA, IAld)	AhR-dependent remodeling → context-dependent p300 (acetylation) or KDM6B/EZH2 (repression)	H3K27ac (barrier) or H3K9me2/3 (inflammatory)	**Hypothalamus:** NF-κB repression, microglial quiescence; **Gut:** *IL22*, tight junctions; **Immune cells:** anti-inflammatory polarization	**Emerging** (immune/neural systems)
**Polyamines** (spermidine, spermine)	Nucleosome stabilization; HAT inhibition; autophagy *via* EP300 inhibition	Chromatin compaction; reduced H3 acetylation	**Multiorgan:** chromatin integrity; **Germline:** meiotic stability, DNA protection; **Somatic gonadal cells:** stress resilience	**Hypothesis-generating** (experimental aging models)
**Pro-inflammatory products** (LPS, flagellin)	NF-κB enhancer activation → persistent H3K27ac/H3K4me1 at inflammatory loci	H3K27ac, H3K4me1 (enhancers)	**Hypothalamus:** trained immunity in microglia; **Liver:** acute phase; **Adipose:** macrophage infiltration; **Gonads:** inflammasome activation	**Established** (immune tissues); **Emerging** (endocrine/reproductive)

Evidence level definitions:

**Established**, direct chromatin assays (ChIP-seq, ATAC-seq, WGBS) in relevant systems.

**Emerging**, preclinical or limited human evidence, often indirect readouts.

**Hypothesis-generating**, primarily associative or correlational findings.

**TABLE 5:** Direct experimental evidence for SCFA-mediated histone acetylation: a tissue-by-tissue summary.

Tissue/ System	Direct evidence?	Techniques used	Key citations	Evidence tier
Intestinal epithelium	**YES**	ChIP-seq (H3K27ac, H3K9ac, H3K27bu, H3K18pr, H3K18bu), WGBS, ATAC-seq	Pan 2018, Gates 2024, Nshanian 2025, Kabir 2025	**Tier 1 – Established**
Immune cells (T cells, B cells, NK cells, mast cells)	**YES**	ChIP-seq (H3K27ac), ATAC-seq	Kespohl 2017, Sanchez 2020, NK/mast cell studies 2025	**Tier 1 – Established**
Hematopoietic stem cells	**YES**	ChIP-seq (H3K27ac, H3K4me1, H3K4me3)	Zeng 2023	**Tier 1 – Established**
**Pancreatic β-cells**	**YES (recent)**	ChIP-seq (H3K27ac) in mouse islets	Pedersen et al., *FEBS J* 2024	**Tier 1 – Emerging**
Colorectal cancer cells (*in vitro*)	**YES**	CUT&Tag, ChIP-seq	Nshanian 2025	**Tier 1 – Established**
**Liver**	**NO** (negative finding)	Mass spectrometry (global histone acetylation)	Saiman et al., *Hepatology* 2021	**Tier 2 – Indirect**
**Hypothalamus/ brain**	**NO**	No ChIP-seq/ATAC-seq in aged hypothalamus; inferred from gene expression and cultured neurons	Mechanistic studies	**Tier 2–3 – Inferred**
**Adipose tissue**	**LIMITED**	Cell culture only; no *in vivo* aged tissue ChIP-seq	Cell culture studies	**Tier 2–3 – Emerging/indirect**
**Gonads (ovary/testis)**	**NO**	No direct chromatin assays; animal models show DNA methylation changes, not histone acetylation	Argaw-Denboba 2024	**Tier 2–4 – Emerging/associative**

### 4.1 Hypothalamus and neuroendocrine aging

As shown in Table 2, SCFAs such as butyrate and propionate cross the blood–brain barrier *via* monocarboxylate transporters (MCT1/2) and accumulate in the hypothalamic arcuate (ARC) and paraventricular (PVN) nuclei. There, they act as natural HDAC inhibitors, helping maintain H3K9ac and H3K27ac at the promoters of POMC, CART, BDNF, and IRS2 (Hoban et al., 2016; Stilling et al., 2014). This open chromatin state supports leptin and insulin sensitivity and limits NF-κB activity (Škandík et al., 2025). As SCFA-producing bacteria such as Faecalibacterium and Roseburia decline with age, histone acetylation at these sites decreases (Gámez-Macías et al., 2024), thereby promoting inflammatory gene expression and microglial activation. Tryptophan-derived indoles (IPA, IAA, IAld) activate the aryl hydrocarbon receptor (AhR) in hypothalamic microglia and astrocytes (Wang Y. et al., 2025; Wei et al., 2021). Under healthy conditions, indole–AhR signaling recruits chromatin remodeling complexes (SWI/SNF) and maintains repressive H3K9me2/3 marks at NF-κB-dependent genes (*IL1B*, *TNF*, *IL6*) (Wang et al., 2026). As people age and indole levels change, these repressive marks decrease, and H3K4me3 increases at these genes, leading to ongoing neuroinflammation. At the same time, age-related endotoxemia causes lasting enhancer remodeling in hypothalamic microglia, as evidenced by increased H3K27ac and H3K4me1 at inflammatory gene enhancers (Erny et al., 2015), This creates an epigenetic state that lowers activation thresholds. The resulting inflammation reduces *GnRH* transcription by promoting histone deacetylation at the promoter, linking microglial chromatin changes to the decline of the hypothalamic–pituitary–gonadal axis (Donati Zeppa et al., 2022; Masri et al., 2015; Schrader et al., 2024). Mechanistic studies have shown that IKK-β and NF-κB block gonadotropin-releasing hormone (GnRH) secretion, leading to a decline in hypothalamic GnRH as people age (Zhang et al., 2013), so treating with GnRH can improve age-related declines in neurogenesis and slow down aging.

#### 4.1.1 Circadian vulnerability adds to these effects

CLOCK–BMAL1 complexes control rhythmic H3K9ac and H3K14ac at clock-controlled genes (Aguilar-Arnal and Sassone-Corsi, 2013), but age-related changes in gut bacteria reduce the rhythmicity of SCFAs and bile acids. This lowers Bmal1 and Per acetylation and disrupts hormone timing (Lee et al., 2024). Outside the central nervous system, SCFA-dependent HDAC inhibition also affects chromatin states in other metabolic organs, starting with the endocrine pancreas. One multi-omic study showed that the microbiome-derived secondary bile acid GDCA can regulate chromatin accessibility in the brain, providing direct evidence for microbiota-brain chromatin signaling (Chakraborty et al., 2025). Although the hypothalamus is involved in many functions that change with age, its exact role in aging remains unclear. Rodent studies have shown that lifespan can be increased or decreased by manipulating the mediobasal hypothalamus, which supports the idea that hypothalamic microinflammation plays a role in aging (Zhang et al., 2013; Mendelsohn and Larrick, 2017). Direct evidence for SCFA-mediated histone acetylation in the brain, especially the hypothalamus, remains very limited (Tier 2–3) and is mostly inferred from gene expression, cell culture, or *ex vivo* studies in mouse brains. The claim that SCFAs “regulate hypothalamic chromatin” (as stated in many reviews, including this one) is based on.

- SCFAs are known to cross the blood–brain barrier through MCT1/2 transporters and build up in hypothalamic nuclei. For example, valeric acid, a five-carbon SCFA, acts as a selective Class I HDAC inhibitor (especially HDAC3) and has been shown to reduce neuroinflammation and provide neuroprotection in the gut–brain axis (Paciolla et al., 2025).
- There is direct evidence of HDAC inhibition in cultured neurons (Tier 3 evidence). For example, SCFAs can enter cells *via* transporters, enter the nucleus, inhibit HDACs, and activate HATs. This leads to increased histone acetylation and altered gene expression (Nshanian et al., 2024).
- Changes in gene expression in the hypothalamus after SCFA or microbiota manipulation show that gut bacteria work with their animal hosts to control the development and function of the immune, metabolic, and nervous systems through ongoing two-way communication along the ‘gut–brain axis’ (Morais et al., 2021). (Tier 2 evidence). There is still a major gap in direct chromatin evidence for the aged hypothalamus.

### 4.2 Metabolic regulation in the pancreas

Pancreatic β-cells rely on stable chromatin structure to release insulin when glucose is present. Table 2 shows that SCFAs help maintain H3K9ac and H3K27ac at key regulatory regions of *INSR*, PDX1, and MAFA (Pan et al., 2021) by blocking HDAC activity. As people age and SCFA levels fall, histone acetylation drops, chromatin tightens, and insulin gene expression decreases. Reduced AMPK activation also raises oxidative stress and causes β-cells to lose their specialized function (Mostafavi Abdolmaleky and Zhou, 2024).

As people get older, endotoxemia triggers NF-κB signaling in pancreatic islets, leading to ongoing production of IL1B, TNF, and interferon-γ (Chen Z. et al., 2022). This inflammation causes lasting changes at cytokine-responsive enhancers, turning short-term immune signals into long-term epigenetic changes (Ramos-Rodríguez et al., 2019). Issues with microbial folate and methionine metabolism lower SAM levels, which makes DNA methylation less stable at genes involved in insulin release, mitochondrial function, and stress responses. When incretin signaling (GLP-1, PYY) is reduced, β-cells face more demand and stress-related genes become more active. Pancreatic β-cell dysfunction does not happen in isolation. The liver, which receives blood rich in microbial metabolites, also shows a similar pattern of chromatin-related metabolic aging.

A 2024 study from the University of Copenhagen provided direct chromatin-level evidence for butyrate in pancreatic islets: butyrate blocked HDAC activity in mouse islets and increased H3 and H4 acetylation by 3- and 10-fold, respectively. Genome-wide H3K27ac ChIP-seq showed that butyrate increased H3K27ac at promoter regions (74%) near hormone secretion genes and decreased H3K27ac near inflammatory genes. The study found that butyrate prevents IL-1β-induced pancreatic islet dysfunction by blocking HDAC and changing H3K27ac levels at genes important for β-cell function (Pedersen et al., 2024; Pedersen et al., 2025). However, more research is needed to confirm these findings. Most current evidence is indirect. For example, *in vitro* and *in vivo* studies of colonic propionate on PYY and GLP-1 release in rodents (Tier 2) show that propionate mainly activates GPR43, while both propionate and butyrate are the strongest activators of GPR41 (Horiuchi et al., 2020; Aragonès et al., 2019) These studies found that propionate and butyrate can increase the release of Peptide YY (PYY) and glucagon-like peptide-1 (GLP-1) in intestinal L cells by activating GPR41 and GPR43, which affects several tissues, including the pancreas and brain. PYY can slow down intestinal movement, reduce appetite, and make people feel fuller (Psichas et al., 2015); GLP-1 mainly helps islet β-cells grow, prevents their death, protects liver cells from fat buildup, and controls blood sugar by improving insulin sensitivity (Qin et al., 2025).

### 4.3 Metabolic aging of the liver

**FIGURE 6:**
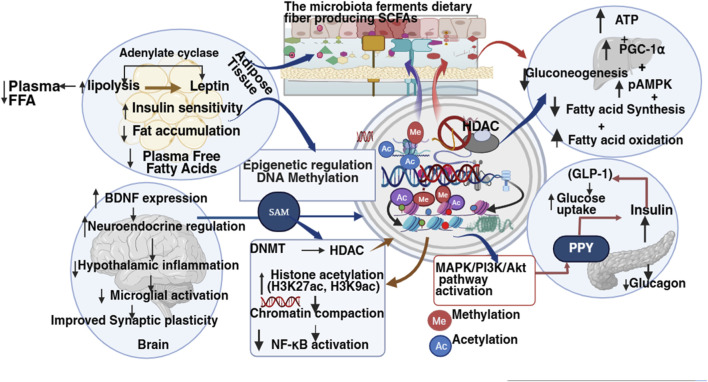
Systemic effects of gut microbiota-derived short-chain fatty acids (SCFAs) on host metabolism and neuroendocrine function. SCFAs, produced by fermentation of dietary fiber, act on multiple organs: **Liver**–Activate AMPK–PGC-1α signaling → enhance fatty acid oxidation, reduce lipogenesis, improve gluconeogenesis and ATP production, mitigate hepatic steatosis. **Pancreas**–Stimulate GLP-1/PYY secretion → activate MAPK/PI3K/Akt → improve insulin secretion, glucose uptake, and glycemic control. **Adipose tissue**–Activate adenylate cyclase, increase leptin, reduce lipolysis and free fatty acids → improve insulin sensitivity and reduce fat accumulation. **Brain**–Increase BDNF, reduce hypothalamic inflammation and microglial activation → improve synaptic plasticity and neuroendocrine stability (e.g., GnRH pulsatility). **Epigenetic regulation**–Modulate SAM availability → alter DNA methylation (*via* DNMT), histone acetylation (H3K27ac, H3K9ac *via* HDAC inhibition), chromatin compaction, and NF-κB activation. These integrated pathways preserve metabolic homeostasis, reduce inflammation, and support neuroendocrine function during aging.

The liver is the first organ to receive gut-derived metabolites and bile acids. As shown in Table 2 and Figure 6, short-chain fatty acids (SCFAs) that reach the liver *via* the portal vein inhibit histone deacetylases (HDACs) and influence DNA methyltransferase (DNMT) activity. This process supports the expression of PPARGC1A (PGC-1α), FOXO1, and HNF4α through both AMPK-dependent and AMPK-independent mechanisms. These transcription factors regulate mitochondrial biogenesis, gluconeogenesis, fatty acid oxidation, and maintenance of hepatocyte identity (Li et al., 2025; He J. et al., 2020). An age-related decline in SCFA-producing gut microbes leads to reduced HDAC inhibition and diminished AMPK activation, resulting in decreased activity of the PGC-1α, FOXO1, and HNF4α regulatory network (Rakshe et al., 2024; Yuan et al., 2016). This shift in hepatic gene regulation promotes increased glucose production, reduced oxidative efficiency, and fat accumulation, which are characteristic features of metabolic inflexibility.

#### 4.3.1 Secondary bile acids

SBA such as deoxycholic acid (DCA) and lithocholic acid (LCA) activate the farnesoid X receptor (FXR), a bile-activated nuclear receptor in hepatocytes (Funabashi et al., 2020). FXR forms a heterodimer with the retinoid X receptor (RXR) and recruits coactivators such as steroid receptor coactivator-1 (SRC-1) and p300 to metabolic enhancers (Sheng et al., 2025). Supporting this, a study found that “in a search for additional cellular targets of p300/CBP, a protein-protein cloning strategy surprisingly identified SRC-1, a coactivator involved in nuclear hormone receptor transcriptional activity, as a p300/CBP interactive protein. p300 and SRC-1 interact, specifically, *in vitro* and they also form complexes *in vivo* (Yao et al., 1996)”. With aging and altered microbial bile acid metabolism, FXR signaling shifts toward reduced enhancer acetylation and impaired repression of inflammatory gene programs. Simultaneously, TGR5 signaling in Kupffer cells reduces NF-κB-dependent chromatin accessibility, thereby restraining transcription associated with the inflammasome (Yang et al., 2021). SRC-1 is essential for the transcriptional activity of FXR and pregnane X receptor (PXR), and its expression declines with age in mouse liver, correlating with impaired bile acid homeostasis (Dawson and Karpen, 2015). Metabolic inflexibility in the liver is paralleled in adipose tissue, where SCFA-dependent chromatin regulation influences the balance between energy storage and inflammatory signaling.

However, direct chromatin immunoprecipitation sequencing (ChIP-seq) or assay for transposase-accessible chromatin sequencing (ATAC-seq) data from hepatocytes following SCFA manipulation in aged animals are currently lacking (Tier 2). As a result, most conclusions are inferred from intestinal or immune cell data or from gene expression analyses. Direct chromatin evidence for SCFA-mediated histone acetylation in the liver remains limited. Most available evidence is indirect and based on mouse models. For example, in one experiment, SCFAs modulated chromatin by inhibiting HDACs, thereby regulating the mTOR pathway and T-cell immune responses that affect liver metabolic inflammation. The H3K27me3 histone marker was increased on promoter regions of inflammatory suppressor genes in mice lacking free fatty acid receptor 2 (FFAR2). Another experiment demonstrated that butyrate reduced HDAC expression and increased Sox17, an inflammatory suppressor gene (Pan P. et al., 2018). Additionally, butyrate has been shown to inhibit HDAC2 expression in the liver, reducing hepatic stellate cell activation and alleviating liver fibrosis in a mouse model of biliary atresia (Zhao et al., 2025) SCFAs influence liver function *via* the gut–liver axis by activating G protein-coupled receptors (GPCRs) such as GPR43 and GPR109A, and by modulating the HDAC1/miR-155 pathway (Li X. et al., 2024).

### 4.4 Adipose tissue remodeling in aging

Adipose tissue works as an endocrine organ, and its ability to adapt depends on chromatin structure. According to Table 2 and Figure 6, SCFAs such as butyrate and propionate help maintain H3K9ac and H3K27ac at PPARG, CEBPA, ADIPOQ, and SLC2A4 (GLUT4) by blocking HDAC activity. This process supports adiponectin release and improves insulin sensitivity, while lowering LEP and RETN expression (Wan et al., 2022; Świderska et al., 2018). Acetate activates free fatty acid receptor 2, which increases leptin release from fat cells, helping to control appetite and reduce obesity (Chambers et al., 2015).

As people get older, they have fewer butyrate-producing bacteria, which lowers histone acetylation at these sites and weakens insulin signaling through IRS1 and SLC2A4. Problems with one-carbon metabolism alter SAM levels, leading to DNA methylation changes at PPARG, IRS1, and SLC2A4 that worsen metabolic issues. Chromatin at UCP1 and PRDM16 becomes harder to access, so brown fat produces less heat (Sakaguchi, 2025). There is some evidence that SCFAs affect histone acetylation in fat tissue, though this remains limited. Butyrate has been shown to lower HDAC9 expression in white fat, thereby promoting browning and heat production in mice with diet-induced obesity (Yang J. et al., 2025); Sodium butyrate (NaBu) increases H3K9 acetylation in stem cells from the periodontal ligament as they become fat cells (Millán-Zambrano et al., 2022); P300-driven histone lysine butyrylation increases during fat cell development, and blocking this process disrupts fat cell formation (Pham et al., 2018). Butyrate affects gene activity in immune cells, such as macrophages, dendritic cells, and Tregs, as well as gut lining cells, by increasing histone acetylation and making chromatin more accessible. This helps reduce inflammation and influences how the body processes fats (Schulthess et al., 2019; Furusawa et al., 2013; Grouls et al., 2022; Chang et al., 2014). Acetate activates free fatty acid receptor 2, increasing leptin release from fat cells. This helps control appetite and may help with obesity (Chambers et al., 2015). Also, propionate causes specific DNA methylation changes in the DAB adaptor protein 1 promoter, a gene linked to diabetes (Guo et al., 2022a). Some theories suggest that SCFA-driven HDAC inhibition might affect the activity of methyl-CpG-binding domain proteins, but this has not been proven. More research using *in vivo* tracers and chromatin profiling is needed. Chronic endotoxemia increases the number of macrophages in fat tissue, elevating levels of IL-6, TNF-α, and IL-1β. When NF-κB is activated, it changes enhancer regions in fat cells, leading to inflammation and blocking genes that support insulin sensitivity (Mayorga-Ramos et al., 2022).

Still, not all age-related changes in fat tissue are due to the microbiota. Aging cells, changes inside fat cells, and shifts in hormones like estrogen and growth hormone can also change chromatin without help from microbes (liver, pancreas, adipose). The hypothalamus, liver, pancreas, and adipose tissue all influence the reproductive system, where germline and somatic gonadal cells have unique epigenetic vulnerabilities to signals from the microbiota. However, there are no direct ChIP-seq or ATAC-seq studies on SCFA-induced histone acetylation changes in aged fat tissue. Most evidence comes from cell culture models (pre-adipocyte cell lines) or young animal models.

### 4.5 Aging of the reproductive system

The reproductive system is highly sensitive to chromatin regulation. DNA stability matters in both hormone-producing somatic cells (granulosa, theca, Leydig, Sertoli) and germ cells (oocytes, spermatogonia). As shown in Table 2, metabolite classes that regulate metabolic tissues also influence gonadal chromatin. This results in distinct effects on fertility and transgenerational inheritance.

#### 4.5.1 Ovary

Aging is characterized by progressive DNA hypermethylation at genes for mitochondrial biogenesis (PGC-1α), meiotic cohesion (SMC1B, REC8), and redox homeostasis (SOD2, GPX4). This is accompanied by erosion of H3K27ac and H3K4me3 and accumulation of H3K9me3/H3K27me3 at steroidogenic genes (CYP19A1, StAR) (Hullar and Fu, 2014; Gleason and Chen, 2023). Epigenetic clock analyses reveal accelerated biological aging in ovarian tissue compared to most somatic organs. During menopause, changes in gut microbiota and estrobolome function are linked to estrogen-related diseases such as breast cancer, endometrial cancer, and polycystic ovarian syndrome (PCOS) (Kumari et al., 2024).

#### 4.5.2 Testis

In spermatogonial stem cells, the balance between H3K4me3 (activation) and H3K27me3 (repression) regulates self-renewal and differentiation (Sheng et al., 2021). With age, disruption of histone-to-protamine exchange leads to abnormal histone retention, increased DNA damage, and altered activation of early embryonic genes (Ded et al., 2024). Studies with germ-free mouse models show that the absence of microbiota impacts male reproductive tract epigenetics across generations (Harris et al., 2023; Barucci et al., 2020). n human studies, age-related changes in testosterone levels correlate with changes in gut microbiota in elderly male patients (Matsushita et al., 2022).

#### 4.5.3 Germline transmission

Short-chain fatty acids (SCFAs) modify acetyl-CoA availability. This influences histone acetylation in gonadal somatic cells. Secondary bile acids and indoles activate nuclear receptors (FXR, AhR), which recruit chromatin remodelers to genes that regulate oxidative stress and mitochondrial function in germline-supporting cells (Epigenetic control of heredity, 2023; Liu et al., 2023). Age-related dysbiosis decreases protective metabolites and increases lipopolysaccharide (LPS) levels. These changes activate NF-κB, STAT3, and p53 signaling in granulosa, Sertoli, and Leydig cells and remodel enhancer landscapes to favor pro-aging gene expression programs (Munyoki et al., 2025).

#### 4.5.4 Extracellular vesicle-mediated signaling

Metabolic and inflammatory stress modify the loading of small RNAs (miRNAs, tRNA fragments) into extracellular vesicles derived from epididymal epithelial cells in males and granulosa cells in females (Chen et al., 2021). These vesicles transfer regulatory RNAs to sperm and oocytes, thereby influencing zygotic gene expression and chromatin remodeling (Liu X. et al., 2024; Kim and Kamada, 2023; Kosan et al., 2018; Jayarajan and Milsom, 2020). In animal models, perturbations of the paternal microbiome alter offspring metabolic health, suggesting the existence of microbiota-dependent germline epigenetic inheritance, although confirmation in humans is currently lacking (Ansari et al., 2020; Lismer and Kimmins, 2023; Ribas-Aulinas et al., 2023; Sun et al., 2024; Wu D. et al., 2024; Takahashi et al., 2023). Depletion of the gut microbiome in male mice is associated with specific changes in sperm small RNA profiles and leads to significant effects on offspring phenotypes (Masson et al., 2025).

#### 4.5.5 Hypothalamic–pituitary–gonadal (HPG) axis integration

Section 4.1 describes how hypothalamic chromatin changes, induced by microbial metabolites, regulate gonadotropin-releasing hormone (GnRH) pulsatility (Bellet and Sassone-Corsi, 2010; Ma et al., 2026). Disruption of GnRH signaling causes secondary chromatin changes in ovarian and testicular cells and establishes a feedback loop between neuroendocrine regulation and gonadal epigenetic stability (Jin et al., 2023). Current evidence comes from animal models, which show that manipulating the microbiota alters DNA methylation in germ cells (Tier 2 evidence). Human observational studies report links between microbiome composition and reproductive hormone levels (Tier 4 evidence) (Argaw-Denboba et al., 2024).

### 4.6 Reproductive epigenetic inheritance: distinguishing animal models from human evidence

This section examines how the microbiota may influence germline cells and contribute to transgenerational inheritance of traits. Evidence from animal models provides experimental proof of causality, whereas human studies primarily yield correlational data. The most robust evidence for microbiota–germline cell interactions comes from controlled animal experiments, mostly in mice.

**Argaw-Denboba et al. (Nature, 2024)** showed that altering the gut microbiota in male mice with non-absorbable antibiotics directly modified the sperm epigenome, including small RNAs and DNA methylation. These epigenetic changes correlated with higher rates of low birth weight, severe growth restriction, and increased early mortality in offspring. The study introduces the ‘gut–germline axis,’ in which changes in the paternal microbiome directly affect placental function and offspring outcomes (Argaw-Denboba et al., 2024).

**Harris et al**. **(bioRxiv, 2023)** reported that in germ-free and antibiotic-treated mice, both the microbiota and the immune system influenced offspring phenotypes independently of genetic alterations. These effects persisted for at least two generations (Harris et al., 2023).

**The Hackett group (EMBO Journal, 2024)** observed that disruption of the gut microbiota in male mice led to reduced expression of genes essential for extra-embryonic tissue development during the blastocyst stage. Additionally, paternal dietary modifications were associated with minor developmental delays (Dura et al., 2025).

**The Florey Institute (Brain, Behavior, and Immunity, 2024)** reported that reducing gut microbiota in male mice using oral antibiotics led to epigenetic changes in sperm. These changes affected the brain development and function of their offspring, leading to lower body weight, changes in gut length, and anxiety-like behaviors in female offspring (Chan et al., 2018).

In humans, the evidence is mostly associative or correlative, and sometimes missing altogether. A recent review made this clear*: “*Despite promising findings, most evidence to link parental diet, gut microbiota and epigenetics to offspring health is from animal studies, highlighting the need for human data (Yang C. et al., 2025) The idea of a “gut–germline axis” is still just a **hypothesis in humans**, supported by animal studies but not yet directly proven as indicated in several studies below; **Zambella et al. (Biology, 2025)** reviewed what is known about seminal microbiota in humans and animals. They concluded: “Despite promising findings, most of the current evidence derives from animal models, and there is a significant lack of human studies.” The review also notes that the composition of the seminal microbiota can be influenced by environmental factors such as diet, chemicals, and lifestyle, but there is currently no direct evidence for microbiota-driven germline epigenetic changes in humans (Zambella et al., 2025). Most studies as indicated below have limited evidecne in humans.

**Senaldi and Smith-Raska (Clinical Epigenetics, 2020)** reviewed evidence for non-genetic inheritance of human traits and found that, while this is well documented in plants, nematodes, and rodents, **evidence in humans remains limited and debated**, with most studies unable to rule out effects from shared environment or culture (Horsthemke, 2018); **Ghai & Kader (Biochemical Genetics, 2021)** reviewed epigenetic inheritance of experiences in humans. They found that animal models help explain mechanisms, but human studies face major challenges in separating true epigenetic inheritance from other factors such as shared diet, socioeconomic status, or parenting behaviors (Ghai and Kader, 2021). The new **MDME (Microbiota-Derived Metabolite-Epigenetic) axis** framework highlights this gap, noting that “most current evidence is derived from model systems, lacking functional validation in humans” and that future research will need “single-cell epigenetics technologies to decipher cell-type specificity (Lei et al., 2025).

This evidence gap does not disprove a role for SCFAs in regulating chromatin in endocrine tissue but highlights the need for further study. Priority areas include: (Borrego-Ruiz and Borrego, 2024): conducting long-term studies that monitor parents’ microbiomes, sperm epigenomes, and child health over time; (Protachevicz et al., 2026); designing studies that test interventions such as probiotic supplementation or dietary changes in prospective fathers and then tracking the health of their children; and (Gyriki et al., 2025) developing non-invasive approaches to measure the epigenetic status of germline cells. Explicitly outlining these research needs will help focus future studies and address current gaps in the evidence.

The table below distinguishes tissues with **direct** chromatin-level evidence (Tier 1: ChIP-seq, ATAC-seq, CUT&Tag, mass spectrometry) from those where evidence is **indirect** (Tier 2–3, inferred from gene expression or cell culture).

## 5 Host-driven mechanisms: bidirectional signaling on how endocrine and metabolic aging remodels the gut microbiota

Organs such as the hypothalamus, pituitary, and gonads, along with the host and microbiota, communicate bidirectionally, forming complex feedback loops that cannot be traced to a single cause. This review has primarily focused on how microbiota-derived metabolites influence endocrine and reproductive aging. It is just as important, though, to examine how aging in the host alters the gut microbiome. The main host-driven factors that alter the gut microbiome during aging are outlined below, showing that dysbiosis can be both a result and a cause of age-related decline. As people age, changes in metabolism can alter the composition and function of the microbiome. Shifts in hormones, immune function, gut physiology, and nutrient levels all create new conditions that influence the gut microbiome, even if the microbes themselves are not directly involved (Nakanishi et al., 2020). The connection between host aging and the microbiota is bidirectional, with each influencing the other (Best et al., 2025; Kim and Benayoun, 2020).

### 5.1 Sex hormone decline and the gut microbiome

As people get older, sex hormone levels change significantly. Women experience a drop in estrogen during menopause, while men see lower testosterone during andropause. These shifts directly affect the gut microbiome.

#### 5.1.1 Estrogen and the gut microbiome

Estrogen receptors are found throughout the digestive tract. Estrogen affects gut barrier function, movement, and immune responses (Maffei et al., 2022). Studies show that postmenopausal women have lower gut microbial diversity and fewer beneficial bacteria, such as *Lactobacillus* and Bifidobacterium, than premenopausal women (Nieto et al., 2025; Peters et al., 2022a). A study of 33 patients with PCOS found higher levels of *Bacteroides*, *Escherichia*/*Shigella*, and *Streptococcus* (Liu et al., 2017). Another study found that women with premature ovarian insufficiency (POI) had gut microbiome diversity similar to premenopausal women with normal ovarian function. However, women with POI had higher abundances of Bacteroidetes, Butyricimonas, Dorea, and Lachnobacterium, and lower abundances of Firmicutes, Bulleidia, and Faecalibacterium (Wu J. et al., 2021; Peters et al., 2022b).

Estrogen replacement therapy can partially restore these changes (Asan-Si). In mice with surgically removed ovaries (a model of surgical menopause), there were major changes in the gut microbiota, including increased *Bacteroides* and decreased Ruminococcaceae, regardless of diet or other interventions (Zeibich et al., 2021; Liu, 2016; Cross et al., 2024). Giving estradiol reversed some of these changes (Wang H. et al., 2025). Recent preclinical and clinical studies show that losing estrogen after menopause can cause problems with the gut-brain axis (GBA). Estrogen deficiency changes the makeup and diversity of the gut microbiome, weakens the gut barrier, and disrupts immune responses involving T cells and microglia in both the gut and the central nervous system (Chaudhary et al., 2026).

#### 5.1.2 Testosterone and microbiome

Testosterone influences the male gut microbiome. In cattle and mice, male castration-induced hypogonadism alters the ileal microbiota. Testosterone replacement restores the original microbial composition (Moadi et al., 2024). Androgens significantly remodel gut microbiota, leading to the interdisciplinary field of the ‘microgenderome.’ More recently, the microbiota has been identified as a major regulator of androgen production and metabolism (Khan et al., 2024). It may also cross the blood-testis barrier (BTB) to regulate spermatogenesis, advancing understanding of male reproduction (Li X. et al., 2022). Gonadectomy, the removal of testicles in males and ovaries in females, combined with hormone replacement therapy, affects the gut microbiota. This intervention also alters mRNA and protein levels of genes linked to the bile acid signaling pathway, mediated by testosterone (Duan et al., 2024). Human studies show that men with age-related hypogonadism and low testosterone have distinct gut microbial profiles, with reduced Akkermansia muciniphila and increased pro-inflammatory taxa (Kaltsas et al., 2025).

### 5.2 Metabolic aging: insulin resistance, bile acid changes, and the microbiome

Beyond hormonal changes, as people age, metabolic problems like insulin resistance, changes in how the body handles fats, and shifts in bile acid production create gut conditions that support specific groups of microbes.

#### 5.2.1 Insulin resistance and gut microbiota

The amount of glucose (a simple sugar) available in the gut. This affects how microbes ferment food. Both insulin resistance and changes in bile acid (molecules that help digest fats) production shift the chemical balance in the gut. These changes influence which microbes thrive there (Best et al., 2025; Rong et al., 2025; Araújo et al., 2024). Mouse studies show that interactions between the body and its gut microbes weaken with age (Best et al., 2025).

Higher levels of Firmicutes and Actinobacteria (types of bacteria), along with lower levels of Bacteroidetes (another type of bacteria), are linked to higher blood LPS (lipopolysaccharide, a component of bacterial cell walls) levels, greater insulin resistance, weight gain, and features of metabolic syndrome (Caricilli and Saad, 2013). Human studies show that older adults with metabolic syndrome have fewer bacteria that produce short-chain fatty acids, such as Faecalibacterium prausnitzii, Roseburia, Dialister, Flavonifractor, Alistipes, *Haemophilus*, and Akkermansia muciniphila (Letchumanan et al., 2022). They also have more pro-inflammatory Enterobacteriaceae (Tamanai-Shacoori et al., 2017; Van Soest et al., 2020). Research suggests that insulin resistance can directly change the makeup of gut bacteria, not just the other way around. People with type 2 diabetes have more Firmicutes and fewer *Bacteroides* in their gut compared to healthy people (Song et al., 2024).

#### 5.2.2 Bile acid metabolism

It also affects gut bacterial enzymes, such as bile salt hydrolase and 7α-dehydroxylase, which modify bile acids (Winston and Theriot, 2020; Fleishman and Kumar, 2024). BAs determine microbiota abundance, diversity, and metabolic activity. Changes in the bile acid pool (primary vs. secondary bile acids) favor certain microbial groups. For example, secondary bile acids, such as DCA and LCA, inhibit some pathogens but promote others. The presence of deoxycholic acid (DCA) in drinking water disrupted the mucosal barrier and caused intestinal inflammation in mice (Liu L. et al., 2018). DCA inhibits mucosal healing, while ursodeoxycholic acid (UDCA) promotes it (Mroz et al., 2018). Additionally, DCA and cholic acid (CA) reduced the expression of α-defensins in cultured ileum *in vitro* (Zhou et al., 2020).

### 5.3 Immunosenescence and inflammaging shape microbiome

As people age, ongoing mild inflammation (“inflammaging”) and a weaker immune system (immunosenescence) occur. These changes directly affect the gut microbiome. Lower IgA production with age means the body is less able to keep harmful bacteria out. This can lead to an overgrowth of pro-inflammatory microbes. Gut aging is also linked to higher risks of GI cancers, constipation, infections, swallowing problems, reflux, and loss of appetite, as well as other issues related to immune decline (Shemtov et al., 2023). Reduced secretion of antimicrobial peptides (e.g., Reg3γ, defensins) alters microbial colonization patterns (Mukherjee and Hooper Lora, 2015; Qi et al., 2025). In aged mice, Reg3β/γ expression is negatively correlated with pro-inflammatory bacteria (Eggerthella and Holdemania) but positively associated with beneficial taxa like Roseburia and Ruminococcus in young mice (Qi et al., 2025). Senescent immune cells secrete pro-inflammatory cytokines (IL-6, TNF-α, IL-1β), altering the gut luminal environment (Jang et al., 2024). In humans, older adults with elevated inflammatory markers (IL-6, CRP) display reduced gut microbial diversity and higher Enterobacteriaceae abundance (Chen et al., 2026; Shintouo et al., 2020). Longitudinal studies show that increases in systemic inflammation precede changes in gut microbiota composition, suggesting inflammation drives dysbiosis (Samson et al., 2022). The bidirectional interactions between host aging and microbiota remodeling establish that Dysbiosis is both a consequence and a driver of age-related endocrine decline.

#### 5.3.1 Controversies and confounding factors

Not all studies agree on how microbiome-derived metabolites regulate histone acetylation and gene regulation related to metabolism and hormones in all endocrine tissues. For example, **Saiman et al. (Hepatology, 2021)** directly tested whether microbiota-derived SCFAs affect liver histone acetylation. Using mass spectrometry, they found that global hepatic histone acetylation remained largely unchanged even after antibiotics reduced SCFAs in the gut and portal vein. However, there were great changes in liver gene expression after microbiota depletion (Saiman et al., 2021). This suggests that the liver may not respond to microbiota-derived SCFAs as directly as intestinal tissues do, or that other mechanisms keep hepatic acetylation stable. Therefore, we should not assume that findings from intestinal studies apply to the liver without direct evidence. As a next step, long-term human studies that combine metagenomics, metabolomics, and epigenome-wide profiling in accessible tissues are needed to fully understand how microbial metabolism affects aging in specific tissues (Rubas et al., 2025).

### 5.4 Gastrointestinal physiological changes with age

Aging exerts direct effects on gut physiology that are independent of microbial influences.

#### 5.4.1 Reduced gut motility

Slower intestinal transit time alters nutrient and metabolite exposure, favoring the growth of distinct bacterial populations. Transit time is the primary determinant of variation in microbial cell counts (Minnebo et al., 2023; Roager et al., 2016). Extended colonic transit time is associated with increased microbial richness and a metabolic shift from carbohydrate fermentation to protein catabolism, as indicated by elevated urinary levels of potentially harmful protein-derived metabolites.

#### 5.4.2 Decreased gastric acid production

Elevated gastric pH permits the survival of ingested bacteria that would otherwise be eliminated (O'May et al., 2005; Tennant et al., 2008). With advancing age, reduced gut motility, altered gastric acid secretion, and changes in gut mucus collectively influence the composition and function of the gut microbiota (Zhang et al., 2023c).

#### 5.4.3 Altered mucus layer

Modifications in mucin composition and thickness influence bacterial adhesion and colonization. The mucus layer serves as a biological niche for the mucus-associated microbiota, which significantly impacts human health (Martens et al., 2018), In aging, *in vitro* models demonstrate that changes in the mucin-adhered bacterial community alter the microbial ecosystem and affect the adhesion capacity of lactobacilli compared to fecal coliforms, bifidobacteria, clostridia, and total anaerobes (Van den Abbeele et al., 2009).

#### 5.4.3 Weakened gut barrier (“leaky gut”)

Increased intestinal permeability allows bacterial products (LPS) to enter the circulation, driving systemic inflammation that further alters the gut environment. Intestinal barrier integrity and its improper functioning can result in the uncontrolled passage of bacterial components, products of bacterial metabolism, and harmful substances, leading to systemic inflammation (Di Vincenzo et al., 2024; Ghosh et al., 2020). Akkermansia may play a key role in maintaining the barrier function of the digestive tract, controlling metabolism and fat storage and its abundance of decreases significantly during aging (Mo et al., 2024). Ongoing, low-level inflammation is common in aging and alters how the body produces antimicrobial peptides and tolerates microbes, potentially allowing more pro-inflammatory microbes to grow (Xu Y. et al., 2024; Xu et al., 2021). Loss of microbiota members who produce compounds like short-chain fatty acids (SCFAs) that bolster the integrity of the gut barrier may help spur inflammaging. A robust barrier keeps microbes and molecules out of the immune-cell-rich underbelly of the gut lining and broader circulation, whereas a leaky barrier can jumpstart an inflammatory response that impacts organs throughout the body (Barron, 2025). In this Animal evidence, aged mice exhibit reduced colonic mucus thickness and increased bacterial-epithelial contact relative to young mice, even under identical housing conditions (Sovran et al., 2019). These changes preced**e** significant alterations in microbial composition, indicating that host physiology may drive microbial shifts. In murine models, administration of exogenous A. muciniphila enhances barrier integrity, reduces endotoxin-induced inflammation, protects against metabolic disorders and inflammatory bowel disease (IBD), and modulates aging processes (Jin et al., 2024). These findings highlight the role of the gut microbiota in aging. A decline in butyrate‐producing bacteria from the Firmicutes phylum, such as Faecalibacterium prausnitzii, Eubacterium rectale, *Clostridium* septum, and Roseburia species (Biagi et al., 2016). This reduction compromised gut integrity and immune function, which is further exacerbated by decreases in secondary bile acids and vitamins (Walrath et al., 2021).

Long-term human studies demonstrate that gut microbiome composition is closely associated with metabolic age and frailty (Wang H. et al., 2024; Ke et al., 2021). Activation of anti-aging properties in the blood of young mice, or dilution of aged mice’s blood plasma, has been shown to promote rejuvenating effects and improve the function of specific tissues. These findings underscore the potential benefits of modulating age-elevated systemic factors, such as senescence-associated secretory phenotypes (SASPs), for rejuvenation therapies (Kim et al., 2022; Ma S. et al., 2022; Mehdipour et al., 2021; Mehdipour et al., 2020; Ximerakis et al., 2023; Zhang B. et al., 2023). Future longitudinal studies tracking hormones, the microbiome, and gene regulation will be necessary to elucidate causal relationships.

The following section, Part III, evaluates the translational and therapeutic implications of these findings, including interventions targeting the microbiota–epigenome axis through fecal microbiota transplantation, dietary modifications, and postbiotic strategies.

Part III: Translational and Therapeutic Implications”.

## 6 Human evidence for FMT-Induced chromatin changes: current status and limitations

Microbiota–epigenetic interactions primarily restore metabolite-driven chromatin networks that help maintain gene expression stability as we age, rather than altering the entire microbial community. Fecal Microbiota Transplantation (FMT) is a promising method that can affect a person’s epigenetic state. Recent research suggests FMT may change DNA methylation and histone patterns. However, it is important to know if there are human studies, not just mouseor other animal studies, that show lasting chromatin changes after FMT. We also need to determine whether these changes have been directly measured using chromatin assays such as ChIP-seq, ATAC-seq, or methylation arrays, or whether they are inferred. Another important question is how long these changes last after treatment. Some studies have directly measured DNA methylation changes in humans after FMT.

For instance**, van der Vossen et al. (Gut Microbes, 2021)** studied 22 people with metabolic syndrome and found that allogenic FMT caused measurable changes in DNA methylation in peripheral blood mononuclear cells (PBMCs) 6 weeks after treatment. These changes were associated with donor microbial engraftment and improved metabolic health. However, the study did not look at effects beyond 6 weeks, and PBMCs are immune cells, not endocrine or reproductive tissues (van der Vossen et al., 2021). Similarly, **Stols-Gonçalves et al. (Gut Microbes, 2023)** examined 21 people with non-alcoholic fatty liver disease (NAFLD) who received repeated vegan allogenic FMT over 8 weeks. Liver biopsies showed changes in DNA methylation, especially at TARS (Threonyl-tRNA Synthetase 1) and ZFP57 (Zinc Finger Protein 57) (Stols-Gonçalves et al., 2023). Multi-omics analysis found links between certain microbial changes (Eubacterium siraeum, Blautia wexlerae) and altered methylation at these sites (Stols-Gonçalves et al., 2023). This is the only human study to date to examine FMT-induced epigenetic changes in a solid organ (the liver). In addition, **Zhang et al. (Journal of Autoimmunity, 2023)** studied 42 patients with systemic lupus erythematosus and found that FMT induced DNA methylation changes in PBMCs that were associated with clinical improvement. Measurements were taken at 12 weeks, but long-term effects were not assessed (Zheng et al., 2023; Horton et al., 2024).

Even with these promising results, important gaps remain. No human FMT studies have used direct chromatin accessibility assays. So far, all studies have used DNA methylation arrays that focus on specific CpG sites, not ChIP-seq or ATAC-seq. This means changes in histone modifications or chromatin accessibility have not been measured in humans after FMT. In mice, transferring the microbiota from old animals to young mice promotes inflammaging, suggesting the microbiota plays an active role. On the other hand, transferring microbiota from young mice to old mice can reverse or reduce some of the metabolic and immune effects of aging (Barron, 2025; Parker et al., 2022; Caetano-Silva et al., 2024). Addressing this, using methods such as ATAC-seq, ChIP-seq, or whole-genome bisulfite sequencing, to directly measure chromatin changes after microbiome interventions would provide stronger evidence for cause and effect. Furthermore, there is currently no data on FMT-induced chromatin changes in human endocrine or reproductive tissues, such as the hypothalamus, pancreas, adipose tissue, or gonads. So far, evidence is limited to blood (PBMCs) and liver samples. Regarding duration, it is not yet known how long these epigenetic changes persist. Most studies have only measured changes 6–12 weeks after FMT. There are no long-term follow-up studies lasting 1 year or more to determine whether these changes persist, decrease, or change over time. Additionally, important differences in species should be considered. For example, mouse studies such as those by Zeng et al. (2023) have shown that heterochronic FMT can reverse age-related changes in histone marks (H3K27ac, H3K4me1, H3K4me3) in hematopoietic stem cells. However, we should be cautious about applying these findings directly to human endocrine tissues (Signal et al., 2024).

Clinically, FMT has been successful in treating stubborn Clostridioides difficile infections. However, its use for other health conditions, especially those less clearly defined than *C. difficile* infection, is still mostly experimental or speculative (Barron, 2025). Current human studies indicate that fecal microbiota transplantation (FMT) can induce measurable changes in DNA methylation in blood immune cells and liver tissue. However, direct human evidence that FMT reverses “epigenetic drift” or produces lasting chromatin alterations in endocrine or reproductive tissues is still lacking. Future research should employ direct chromatin assays (e.g., ChIP-seq, ATAC-seq) (Hou et al., 2024), include long-term follow-up (≥1 year), and focus on endocrine and reproductive tissues in appropriate patient cohorts. Investigators should also determine whether FMT effects are tissue-specific or systemic. Additionally, donor selection should prioritise microbial functional capacity (Bibbò et al., 2020), and studies should track microbial persistence and long-term gene regulation, particularly in hormone-sensitive or reproductive tissues.

### 6.1 Dietary and pro-postbiotic modulation of microbiota–epigenetic signaling

Dietary patterns modulate the types and abundances of gut microbes, which, in turn, affect the production of compounds that regulate the structure of host DNA (chromatin). Prebiotics are dietary fibers that stimulate beneficial microbes, while postbiotics consist of microbial metabolites or heat-inactivated microbes. Both can restore the balance of chemical modifications on histones (such as acetylation) without introducing live microbes into the body.

Animal studies show that high-fat, low-fiber Western diets reduce gut microbial diversity and deplete SCFA-producing bacteria (Li D. et al., 2022; Zhu et al., 2021), while fiber-rich diets promote SCFA production. In aged mice, supplementation with inulin or resistant starch increased cecal butyrate and propionate, which correlated with elevated H3K9ac and H3K27ac in colonic epithelium and reduced NF-κB-dependent inflammatory gene expression (Han et al., 2023). Fiber-driven SCFA restoration also stabilized hypothalamic chromatin and reduced neuroinflammation *via* TET-dependent DNA demethylation (Xu et al., 2026). In humans, randomized controlled trials demonstrate that high-fiber regimens, such as the Mediterranean diet, increase fecal SCFA concentrations and alter microbiota profiles (Kimble et al., 2023; Wang et al., 2021). Individuals aged 65–79 years who adhered to a Mediterranean diet for 1 year showed improved cognitive function and lower inflammatory and frailty markers; their microbiomes were enriched for SCFA production (Barron, 2025). However, direct chromatin changes in endocrine or reproductive tissues following dietary fiber interventions have not been assessed in humans. While the Mediterranean diet reduces cardiometabolic disease risk and may work through microbiota-derived indoles and SCFAs, causality and specific epigenetic mechanisms remain unproven.

### 6.2 Postbiotics: direct administration of microbial metabolites

Unlike probiotics, postbiotics circumvent the requirement for live microbes by delivering defined, dose-controlled molecular signals. (SCFAs), including butyrate, propionate, and acetate, are key examples of these postbiotic metabolites. (SCFAs) as natural inhibitors of (HDACs) (Section 3.3.1), maintain open chromatin at metabolic and at metabolic and hormone responsive genec to be more easily activated. For animal evidence, in HFD-fed mice, oral butyrate or tributyrin (a butyrate prodrug) improved insulin sensitivity, reduced hepatic steatosis, and lowered inflammatory enhancer activity in adipose tissue (Arnoldussen et al., 2017; Weng et al., 2015).

In humans, oral butyrate supplementation has yielded inconsistent results: two small trials reported improved insulin sensitivity, whereas other studies found no effect (Bouter et al., 2018). According to a 2024 meta-analysis, “evidence for oral SCFA supplementation in humans remains inconclusive due to high heterogeneity and short treatment durations” (Magrin et al., 2020), EFSA Journal, 2024). Importantly, there are no human trials assessing chromatin changes after SCFA administration.

In addition to SCFAs, indole derivatives activate the aryl hydrocarbon receptor (AhR), recruiting chromatin remodeling complexes to immune and metabolic enhancers (Section 3.5.2). Animal evidence demonstrates that in aged mice, dietary tryptophan or *Lactobacillus* strains producing IPA reduced hypothalamic inflammation, stabilized GnRH expression, and maintained HPG axis function (Xu et al., 2025; Mossad et al., 2022). In humans, lower circulating indole levels are associated with higher inflammatory markers and poorer metabolic health in older adults (Pan et al., 2024). However, no interventional human study has tested whether indole supplementation improves endocrine aging or alters chromatin.

### 6.3 Probiotics: live microbes with epigenetic potential

Turning to probiotics, the effects are strain-specific and frequently context-dependent. The majority of evidence for epigenetic modulation by probiotics comes from animal models. Furthermore, short-chain fatty acids have demonstrated anticancer effects in colorectal, gastric, breast, prostate, and liver cancers (Alrubaye et al., 2019). SCFAs have been shown to have anti-cancer effects in colorectal, gastric, breast, prostate, and liver cancers (Mirzaei et al., 2021).

#### 6.3.1 Lactobacillus rhamnosus GG (LGG)

In rats fed a high-fat diet, LGG supplementation prevents high-fat diet-induced DNA hypermethylation at the peroxisome proliferator-activated receptor gamma (PPARγ) promoter in fat tissue and reduces the transfer of metabolic dysfunction to offspring (He M-Q. et al., 2020). These effects were linked to changes in blood metabolites that influence histone acetylation and nuclear receptor signaling. No human studies have yet looked at LGG’s effects on these specific epigenetic markers. Akkermansia muciniphila, given live or pasteurized to high-fat diet-fed mice, improved insulin resistance, reduced fat tissue inflammation, and increased cecal propionate and butyrate. These changes lowered HDAC activity in fat tissue (Raftar et al., 2022). In a randomized double-blind trial in overweight/insulin-resistant volunteers, pasteurized A. muciniphila for 3 months significantly improved insulin sensitivity, reduced plasma insulin, and lowered total cholesterol (Depommier et al., Nature Medicine, 2019) (Yang L. et al., 2025). Notably, the effect was observed with heat-killed bacteria, indicating a postbiotic mechanism (Kawaguchi et al., 2025). However, no chromatin measurements were performed. A follow-up study confirmed safety and tolerability but did not assess epigenetic endpoints.

#### 6.3.2 Multi-strain probiotics

Animal evidence shows that a multi-strain mixture (containing *Lactobacillus*, Bifidobacterium, and *Streptococcus*) improved adiposity, insulin resistance, and dyslipidemia in HFD-fed mice through adipose tissue immune cell remodeling. The mixture reduced M1 macrophages and increased M2 (Chen A-C. et al., 2022; Tchamani and Yako, 2026). It restored GPR43 expression (an SCFA receptor) and increased Akkermansia muciniphila abundance. *In vitro*, the mixture also enhanced butyrate and propionate production (Alard et al., 2016). Human evidence from meta-analyses of multi-strain probiotics in metabolic syndrome shows modest improvements in fasting glucose and insulin, but results are inconsistent (Mederle et al., 2024). No human trial has linked probiotic supplementation to direct changes in chromatin.

### 6.4 Engineered microbes and next-generation approaches

To address the differences and unpredictability of natural probiotics, researchers are engineering microbes to produce precise regulatory molecules, such as butyrate and indole. For example, a modified Lactococcus lactis strain that continually makes butyrate has been shown to reduce inflammation in mouse models of gut disease (Rozanov et al., 2025). In humans, these methods have only been tested in preclinical settings. For regulatory approval, it will be necessary to prove safety, including showing no exchange of genetic material or antibiotic resistance, and to ensure long-term stability of the engineered microbes (Meng et al., 2026; Arukha et al., 2021). Despite progress, key gaps and future directions remain. First, no human trial has directly measured chromatin changes (ChIP-seq, ATAC-seq, or genome-wide methylation arrays) after any dietary, probiotic, or postbiotic intervention in endocrine or reproductive tissues. Second, most human studies rely on metabolic endpoints (insulin, glucose, lipids) and associate these with microbiota changes, but do not prove epigenetic causation. Third, durability is unknown even when metabolic benefits are observed (e.g., A. muciniphila for 3 months) long-term persistence of any epigenetic effect has never been assessed. Fourth, tissue specificity is ignored; almost all human studies measure blood or fecal markers rather than the hypothalamus, pancreas, adipose tissue, or gonads.

## 7 Future directions: closing the gap between association and causation

In this review, we found several gaps in the evidence. Most human data remain associative (Tier 4). There is still a shortage of direct chromatin assays, such as ChIP-seq, ATAC-seq, and WGBS, for endocrine and reproductive tissues. We do not know how long microbiota-induced epigenetic changes last in humans. The causal relationship between dysbiosis and endocrine decline remains uncertain. Most evidence comes from cross-sectional studies of gut microbes, circulating metabolites, and DNA methylation clocks (Tier 4). These studies cannot show whether dysbiosis causes epigenetic aging or if the reverse is true. To address this, researchers should follow participants in FMT (fecal microbiota transplantation) or probiotic trials for at least 1 year. They should use repeated epigenetic profiling with DNA methylation arrays and, when possible, ATAC-seq on accessible cells such as PBMCs (peripheral blood mononuclear cells) (Morandini et al., 2023; Rechsteiner et al., 2022). This approach will help determine if epigenetic changes are temporary or lasting. Interventional studies with washout periods can test if changes are reversible. For example, (FMT) have been tested for whether it could provide the clinical benefit of alleviation of NAFLD. FMT is the latest mode of gut microbiota manipulation for the treatment of NAFLD (Hauser et al., 2025; Qiu et al., 2024), so when possible, tissue-specific durability should also be assessed. For instance, analyzing liver biopsies from NAFLD patients before and 12 months after FMT can directly track methylation changes in a metabolic organ. To date, the vast majority of these methods have only been tested *in vitro* or animal models. Future research should address these issues to advance the field.

### 7.1 Targeting specific epigenetic regulators, not whole microbiome

Recommended actions are as follows: (Borrego-Ruiz and Borrego, 2024): Run clinical trials of defined postbiotics such as delayed-release butyrate, tributyrin, or spermidine, using clear epigenetic endpoints. Recently, Beteri et al. (2024) utilised both galactopolysaccharides and exopolysaccharides from Bifidobacterium breve postbiotics in a clinical trial with prediabetic volunteers and reported a significant increase in butyrate-producing bacteria, indicating its potential in managing prediabetes with minimal invasive intervention (Beteri et al., 2024). Similarly, gamma-aminobutyric acid (GABA)-rich postbiotics improved metabolic indices in diabetic mice models (Abdelazez et al., 2022). Essentially, the goal would be to craft microbiome therapies tailored to an individual’s microbial composition, a facet of precision medicine. This requires extensive microbial genomic analysis and safety testing (Protachevicz et al., 2026). Optimize dosing, pharmacokinetics, and tissue distribution for these interventions (Gyriki et al., 2025). Test engineered live biotherapeutics products (eLBPs) that produce a single metabolite, such as butyrate-producing Lactococcus lactis in preclinical and early human studies to assess safety and epigenetic effects (Rutter et al., 2022). In a more recent example, **Cubillos-Ruiz et al. (Nature Biomedical Engineering, 2022)**, engineered Lactococcus lactis to produce a heterodimeric β-lactamase, showing that the strain was able to reduce ampicillin-induced dysbiosis in a mouse model (Cubillos-Ruiz et al., 2022). This is where using eLBPs offers several advantages over the use of pre-, pro-, or synbiotics as genetic engineering can confer functions that are not expressed by the native microbiota (Cubillos-Ruiz et al., 2022). Also in a ground-breaking study, Steidler et al. (2000) demonstrated this principle by engineering Lactococcus lactis to produce interleukin-10 for the treatment of murine colitis (Beteri et al., 2024). To date, the vast majority of these methods have only been tested *in vitro* or animal models, and the major issue is that positive *in vitro* and animal model data do not always translate into safe and efficacious treatment in humans. With further development of these new technologies and research, these therapies will be available by translating some of the many developed eLBP strains into clinical treatments (Morandini et al., 2023). Evaluate combinations of a metabolite (e.g., butyrate) with a histone deacetylase inhibitor or a methyl donor (e.g., folate) in animal models to determine whether they stabilize chromatin before progressing to human studies.

### 7.2 Resolving the directionality of the microbiota–endocrine axis

As discussed in Section 5, several host-driven factors can reshape the microbiome. This includes declining sex hormones, insulin resistance, aging of the immune system, and changes in gut function. Most intervention studies still focus on how the microbiota affects the host, rather than the reverse. Recommended actions include using cross-lagged panel analysis in longitudinal cohorts to compare the effects of host aging on the microbiota and *vice versa*. At least three time points and appropriate models are needed. We should think about aging-microbiome interactions as a 2-way street. On the one hand, gut bacterial community changes as the host ages. As a result, the overall metabolism by this community changes as well. On the other hand, the gastrointestinal tract from an aging host can become more susceptible to inflammation and tissue damage at an older age, thereby no longer serving as a nourishing environment for the same gut bacterial community that was present in their youth.” Shuo Han, Ph.D., Duke University (Barron, 2025).

Researchers should also run intervention studies that target host factors, such as estrogen replacement in postmenopausal women or testosterone in hypogonadal men. These studies should include profiling of the microbiome and epigenome before and after. If hormone restoration reverses dysbiosis and epigenetic changes, this would support a host-driven model. M**echanistic** animal experiments with ovariectomized or castrated aged mice, with and without hormone replacement, can help map the causal chain from hormone loss to gut changes, microbiome shifts, and altered chromatin in endocrine tissues.

### 7.3 From animal models to human reproductive epigenetic inheritance

As mentioned in Section 4.6.1, the ‘gut–germline axis’ is well established in mice but remains a hypothesis in humans. So far, no controlled human study has shown that changing the parental gut microbiota directly affects children’s epigenetics or health. Here are some r**ecommended actions:**

Researchers should conduct prospective cohort studies in couples planning pregnancy. They should collect fecal, blood, and, for males, semen samples before conception. Researchers should then follow the offspring’s health. Sperm small RNA and DNA methylation profiles should be correlated with the paternal microbiome and child outcomes. Interventional pilot studies in men undergoing fertility treatment are also recommended. Development of microbiota-based diagnostic tools or biomarkers for male infertility like a simple stool test, might help identify men whose infertility is related to dysbiosis. For instance, a “high endotoxin-producing microbiome” signature or low diversity could flag individuals who would benefit from microbiome-targeted therapies. Participants can be randomized to a probiotic or dietary intervention for 3 months before semen analysis.

Researchers should assess the sperm epigenome (small RNAs, DNA methylation) and, if possible, embryo development. Mendelian randomization analyses have been attempted (using genetic proxies for microbiome traits to infer causality), and some suggest a causal influence of certain gut bacteria on infertility risk (Wang and Kang, 2025; Fu et al., 2023), but these are preliminary. Controlled trials (e.g., giving a prebiotic *versus* a placebo for a year to men at risk of infertility) could robustly demonstrate causation. Additionally, metabolite profiles in blood (like high TMAO or low butyrate levels) could be proxies for gut microbial function and correlate with fertility status (Rahman et al., 2023).

Overinterpretation of the current evidence should be avoided. Claims regarding “transgenerational epigenetic inheritance *via* the microbiome” in humans remain hypothesis-generating in the absence of interventional or well-controlled longitudinal studies. Establishing causality would require, for example, randomized trials in which prospective parents receive a defined microbiota-modulating intervention, such as a live biotherapeutic, and offspring are monitored for epigenetic and health outcomes. Until such data are available, all inferences should be explicitly qualified. For instance, if Faecalibacterium prausnitzii is confirmed to support spermatogenesis through anti-inflammatory or epigenetic mechanisms, such as SCFA-mediated HDAC inhibition in Sertoli cells, a live biotherapeutic product containing this strict anaerobe could be developed (Martín et al., 2023; Kaltsas et al., 2025). However, product development must be followed by rigorous evaluation in well-defined patient populations, such as men with idiopathic oligozoospermia and low faecal F. prausnitzii abundance, with clearly defined endpoints including changes in sperm DNA methylation, histone retention patterns, pregnancy rates, and offspring health. Mechanistic studies should also confirm that the therapeutic effect is mediated by the proposed epigenetic pathway rather than by secondary metabolic or immune changes. Only through such phased, evidence-driven translation can the field progress from association to clinically actionable interventions. When asked about the future of the field, Han pointed to identifying gut bacterial products (e.g., metabolites) and deciphering how they contribute to distinct aspects of host aging biology, such as brain and metabolic health. Most metabolites bacteria make are unknown or understudied, meaning what they do is largely a mystery (Barron, 2025).

## 8 Conclusion

The gut microbiota-epigenome axis demonstrates that intestinal microbes and their metabolites can influence aging. These effects are seen especially when chromatin in endocrine and reproductive tissues is altered. Most current evidence comes from animal studies and some human associations. These studies suggest that microbiota metabolites—such as short-chain fatty acids, secondary bile acids, tryptophan indoles, and polyamines—can influence DNA methylation, histone acetylation, and nuclear receptor signaling. Still, direct evidence that microbiome changes cause endocrine decline in humans is limited and remains unconfirmed.

As people age, they often lose bacteria that produce short-chain fatty acids. This loss leads to weaker HDAC inhibition and less histone acetylation at genes involved in metabolism and steroid response. At the same time, increased endotoxemia and chronic inflammation activate NF-κB. These changes alter enhancer regions and disrupt hormone signaling between the brain and reproductive organs. On the other hand, restoring a healthy microbiome has shown positive effects in animal studies and some human trials. This includes high-fiber diets, postbiotics such as butyrate and spermidine, Akkermansia muciniphila, or possibly fecal microbiota transplants. These approaches can improve insulin sensitivity, reduce inflammation, and help maintain chromatin accessibility.

To move from finding associations to establishing causation, researchers require robust experimental designs and advanced multi-omic tools. These include long-term metagenomics, metabolomics, and direct chromatin assays such as ChIP-seq and ATAC-seq on human tissues. Future studies should emphasize well-controlled trials with at least 1 year of follow-up. Trials should also include washout periods to evaluate the persistence of observed effects and group patients by their initial microbiome and epigenetic profiles. The goal of creating diagnostic tools based on the microbiome, such as fecal SCFA profiles or microbial age clocks, is to enable more precise targeting of the epigenome; however, the causal relationships between these biomarkers and epigenetic changes remain to be established. Testing personalized treatments—such as specific postbiotics or engineered live biotherapeutics may provide insight into these potential causal pathways. While studies in animals demonstrate that epigenetic changes can be inherited by subsequent generations, whether this occurs in humans is unproven and will require longitudinal studies that follow both parents and children.

A deeper understandring of the bidirectional gut microbiota–epigenome axis offers a vital chance to addess age-related endocrine and reproductive decline. Achieving this will not only deepen insights into aging biology but also advance the field. It will also open the door to precision interventions that refine preventive and therapeutic strategies to support healthier, longer lives.

## Glossary

SCFAs: Short-chain fatty acids
HDAC: Histone deacetylase
DNMT: DNA methyltransferases
HMT: Histone methyltransferases
H3K27ac: Histone H3 Lysine 27
H3K4me3: Histone H3 Lysine 4 Trimethylation
BDNF: Brain-derived neurotrophic factor
SWI/SNF: SWItch/Sucrose Non-Fermentable chromatin remodeling complexes
AhR: Aryl hydrocarbon receptor
GPCR: G-protein–coupled receptor
SIRT: Sirtuin
CLOCK: Circadian Locomotor Output Cycles Kaput
BMAL1: Basic helix-loop-helix ARNT–like protein
PDX1: Pancreatic Duodenal homebox1
MAFA: Musculoaponeurotic firbrosarcoma oncogene homolog A
ISN: Insulin gene
FOXO1: Forkhead Box protein O1 gene
HNF4α: Hepatocyte Nuclear Factor 4α gene
CBP: CREB-binding protein
LCA: Lithocholic Acid
TCA: Tricarboxylic Acid (Cycle)
FXR: Farsenoid X receptor
RXR: Retinoid X Receptor
SRC-1: Steroid Receptor co-activator one
TGR5: Tekada G protein-coupled receptor 5
IRS1or 2: Insulin Receptor Substrate one and 2
TMAO: Trimethylamine N-oxide
SLC2A4: Solute Carrier Family 2 Member 4
UCP1: Uncoupling protein one
PPARγ: peroxisome proliferator–activated receptor gamma
C/EBPα: CCAAT enhancer-binding protein alpha
PPARGC1α: peroxisome proliferator–activated receptor gamma coactivator-1 alpha
PRDM16: PR domain-containing protein 16
POMC: Pro-opiomelanocortin
CART: Cocaine- and amphetamine-regulated transcript peptide
ADIPOQ: Adiponectin
LEP: Leptin
RETN: Human resistin gene
sncRNA: Small non-coding RNA
PI3K–AKT: Phosphoinositide 3-Kinase
FXR: Farnesoid X Receptor
IL-6: Interleukin-6
LPS: Lipopolysaccharide
GPCR: G-Protein-Coupled Receptor
GPBA: G Protein-Coupled Bile Acid Receptor
SAM: adenosylmethionine
SAH: S-adenosylhomocysteine
MAPK: Mitogen-Activated Protein Kinase
HPA axis: Hypothalamic–pituitary–adrenal axis
NF-κB: Nuclear Factor Kappa B
STAT3: Signal Transducer and Activator of Transcription
TNF-α: Tumor Necrosis Factor Alpha
HAT: histone acetyltransferase
HP1α: heterochromatin protein one alpha
NuRD: nucleosome remodeling and deacetylase complex
ChIP-seq: chromatin immunoprecipitation sequencing
ATAC-seq: assay for transposase-accessible chromatin sequencing
FMT: Fecal Microbiota Transplantation
NAD+: Nicotinamide adenine dinucleotide
DCA: Deoxycholic Acid
DNA: Deoxyribonucleic Acid
MiRNA: MicroRNA
LncRNA: Long non-coding RNA
